# Targeting histone deacetylase-3 blocked epithelial-mesenchymal plasticity and metastatic dissemination in gastric cancer

**DOI:** 10.1007/s10565-021-09673-2

**Published:** 2022-01-01

**Authors:** Sheng-Mao Wu, Yee-Jee Jan, Shih-Chuan Tsai, Hung-Chuan Pan, Chin-Chang Shen, Cheng-Ning Yang, Shu-Hua Lee, Shing-Hwa Liu, Li-Wei Shen, Chien-Shan Chiu, Jack L. Arbiser, Menghsiao Meng, Meei-Ling Sheu

**Affiliations:** 1 Institute of Biomedical Sciences, College of Life Sciences, National Chung Hsing University, Kuo Kuang Road, 250 Taichung, Taiwan; 2 Department of Pathology and Laboratory Medicine, Taichung Veterans General Hospital, Taichung, Taiwan; 3 Department of Nuclear Medicine, Taichung Veterans General Hospital, Taichung, Taiwan; 4 Department of Neurosurgery, Taichung Veterans General Hospital, Taichung, Taiwan; 5 Graduate Institute of Biotechnology, National Chung Hsing University, Taichung, Taiwan; 6 Institute of Nuclear Energy Research, Atomic Energy Council, Taoyuan, Taiwan; 7 Department of Dentistry, School of Dentistry, College of Medicine, National Taiwan University, Taipei, Taiwan; 8 Institute of Toxicology, College of Medicine, National Taiwan University, Taipei, 100 Taiwan; 9 Department of Medical Research, China Medical University Hospital, China Medical University, Taichung, Taiwan; 10 Department of Dermatology, Taichung Veterans General Hospital, Taichung, Taiwan; 11 Department of Dermatology, Emory University School of Medicine, Winship Cancer Institute, Atlanta Veterans Administration Health Center, Atlanta, GA USA; 12 Department of Medical Research, Taichung Veterans General Hospital, Taichung, Taiwan; 13 Ph.D. Program in Translational Medicine, Rong Hsing Research Center for Translational Medicine, National Chung Hsing University, Taichung, Taiwan

**Keywords:** Histone deacetylase inhibitors, Histone deacetylase 3, Peritoneal dissemination, Epithelial–mesenchymal transition, Endoplasmic reticulum stress

## Abstract

**Background and purpose:**

Histone deacetylase (HDAC) inhibitors (HDIs) can modulate the epithelial-mesenchymal transition (EMT) progression and inhibit the migration and invasion of cancer cells. Emerging as a novel class of anti-cancer drugs, HDIs are attracted much attention in the field of drug discovery. This study aimed to discern the underlying mechanisms of Honokiol in preventing the metastatic dissemination of gastric cancer cells by inhibiting HDAC3 activity/expression.

**Experimental approach:**

Clinical pathological analysis was performed to determine the relationship between HDAC3 and tumor progression. The effects of Honokiol on pharmacological characterization, functional, transcriptional activities, organelle structure changes, and molecular signaling were analyzed using binding assays, differential scanning calorimetry, luciferase reporter assay, HDAC3 activity, ER stress response element activity, transmission electron microscopy, immune-blotting, and Wnt/β-catenin activity assays. The in vivo effects of Honokiol on peritoneal dissemination were determined by a mouse model and detected by PET/CT tomography.

**Key results:**

HDAC3 over-expression was correlated with poor prognosis. Honokiol significantly abolished HDAC3 activity (Y298) via inhibition of NFκBp65/CEBPβ signaling, which could be reversed by the over-expression of plasmids of NFκBp65/CEBPβ. Treatments with 4-phenylbutyric acid (a chemical chaperone) and calpain-2 gene silencing inhibited Honokiol-inhibited NFκBp65/CEBPβ activation. Honokiol increased ER stress markers and inhibited EMT-associated epithelial markers, but decreased Wnt/β-catenin activity. Suppression of HDAC3 by both Honokiol and HDAC3 gene silencing decreased cell migration and invasion in vitro and metastasis in vivo.

**Conclusions and implications:**

Honokiol acts by suppressing HDAC3-mediated EMT and metastatic signaling. By prohibiting HDAC3, metastatic dissemination of gastric cancer may be blocked.

**Graphical abstract:**

Conceptual model showing the working hypothesis on the interaction among Honokiol, HDAC3, and ER stress in the peritoneal dissemination of gastric cancer. Honokiol targeting HDAC3 by ER stress cascade and mitigating the peritoneal spread of gastric cancer. Honokiol-induced ER stress–activated calpain activity targeted HDAC3 and blocked Tyr298 phosphorylation, subsequently blocked cooperating with EMT transcription factors and cancer progression. The present study provides evidence to demonstrate that HDAC3 is a positive regulator of EMT and metastatic growth of gastric cancer cells. The findings here imply that overexpressed HDAC3 is a potential therapeutic target for honokiol to reverse EMT and prevent gastric cancer migration, invasion, and metastatic dissemination.

• Honokiol significantly abolished HDAC3 activity on catalytic tyrosine 298 residue site. In addition, Honokiol-induced ER stress markedly inhibited HDAC3 expression via inhibition of NFκBp65/CEBPβ signaling.

• HDAC3, which is a positive regulator of metastatic gastric cancer cell growth, can be significantly inhibited by Honokiol.

• Opportunities for HDAC3 inhibition may be a potential therapeutic target for preventing gastric cancer metastatic dissemination.
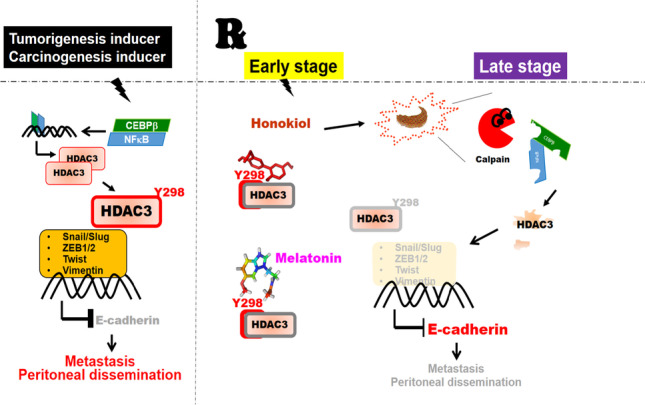

**Supplementary Information:**

The online version contains supplementary material available at 10.1007/s10565-021-09673-2.

## Introduction

The incidence and mortality of cancers, including gastric cancer, are linked with tumor progression, from localized primary tumors to more advanced stages that metastasize and involve multiple organs (Brabletz 2012; Gherardi et al. 2012; Williams et al. 2019). In cancers of the gastrointestinal tract, the peritoneum is the primary site of metastasis. The spread is associated with the loss of epithelial characteristics and the acquisition of a mesenchymal phenotype, leading to invasion and metastasis (Brabletz et al. 2018). Gastric cancer is the second leading cause of cancer-related deaths globally (GBD 2017 Stomach Cancer Collaborators 2020). Most patients will relapse or have local recurrence after definitive surgical treatment. Moreover, patients with gastric cancer have a high risk of lymph node metastases and poor prognosis due to extensive invasion (Juttner et al. 2006; Maehara et al. 1999). A major cause of death is the generation of distant metastasis, which places substantial limits to successful therapeutic interventions (Liu et al. 2015; Wu et al. 2016b). The overall 5-year survival rate of patients with advanced gastric cancer even after treatment remains unsatisfactory, without significant improvement in the past decades (Liu et al. 2015; Wu et al. 2016b). Thus, there is an urgent need to investigate the molecular mechanisms of gastric cancer metastasis and identify the novel biomarkers that may predict metastasis.

Recent evidence has shown a close relationship between epithelial-to-mesenchymal transition (EMT) and disease progression (Chiu et al. 2019; Lai et al. 2014; Liu et al. 2015; Wu et al. 2016b). The EMT is considered a key process in the metastatic step that confers fundamental abilities of tumor cell migration, invasiveness, and anoikic resistance, during which non-motile, polarized epithelial cells dissolve their cell-to-cell junctions and are converted into individual, motile mesenchymal cells (Brabletz et al. 2018; Gherardi et al. 2012). Reduction or loss of cell surface E-cadherin is a classic EMT hallmark, while E-cadherin down-regulation has been correlated with short disease-free survival and poor outcomes (Brabletz et al. 2018; Chiu et al. 2019; Wu et al. 2016b).

During tumor metastasis, EMT may give rise to the dissemination of single tumor cells from the primary epithelial tumor. These are disrupted by numerous signaling pathways and regulatory transcriptional networks, and controlled by distinct signaling molecules in the tumor micro-environment (Jung et al. 2015; Tsai and Yang 2013). As it can be regulated by transcription factors, EMT progression requires the robust transcriptional machinery and post-transcriptional mechanisms, which consist largely of developmental transcription factors that regulate and coordinate epithelial and mesenchymal markers (Brabletz et al. 2018; Chiu et al. 2019; Lai et al. 2014; Liu et al. 2015; Wu et al. 2016b). Dissecting the molecular mechanisms that regulate EMT and E-cadherin expression is crucial in understanding tumor invasiveness and metastasis.

Discovered in 2000, class I histone deacetylase (HDAC3) is a predominant HDAC associated with nuclear receptor co-repressor 1 (NCoR1 or NCoR) and the silencing mediator of retinoic acid and thyroid hormone receptor (SMRT, also known as NCoR2) (Mani and Barton 2011). A previous study has demonstrated that hypoxia-induced HIF-1α-mediated activation of HDAC3 occurs in both epithelial and mesenchymal cells and is essential for EMT and metastasis (Wu et al. 2011). In epithelial cells, hypoxia induces HDAC3 expression and its enzymatic activity towards the removal of the acetyl group of histone 3 lysine 4 (H3K4Ac). This facilitates epithelial gene repression, the activation of mesenchymal gene expression, and the repression of the epithelial-associated gene chromatin structure (Mani and Barton 2011). One crucial mechanism is via the interplay between HDAC3 and WDR5 to regulate EMT marker genes such as E-cadherin and vimentin (Wu et al. 2012, 2011). Gene silencing of HDAC3 abolishes the EMT marker gene regulation in response to hypoxia-induced condition (Chen et al. 2016). Thus, HDAC3 plays a decisive role in hypoxia-induced EMT. However, the role of HDACs in EMT is controversial. Several studies reported that HDACs inhibited EMT, but this is not always the case. HDAC inhibitors may reinforce EMT progression in various conditions (Debeb et al. 2012; Ji et al. 2015; Kong et al. 2012; Sakamoto et al. 2016; Wawruszak et al. 2019a, b; Wu et al. 2016a).

All mammalian HDACs contain putative sites of post-translational modification, including phosphorylation sites that may alter HDAC3 activity and stability, or protein complex assembly similar to how many nuclear receptor co-regulators work (Emmett and Lazar 2019). Distinct mechanisms also show Y298F and K25A mutations that abolish the HDAC3 enzymatic activity (Sun et al. 2013). The crystal structure of a eukaryotic zinc-dependent histone deacetylase reveals highly conserved Tyr residues (Y298 in HDAC3) located within the active site. These are catalytically essential in stabilizing the tetrahedral intermediate and in polarizing the substrate carbonyl for nucleophilic attack in coordination with the Zn ion. The mutation of Y298F (YF) causes the complete inactivation of in vitro–translated HDAC3 proteins. More importantly, their interactions with a number of miRNAs or transcription factors may block the HDAC3-regulated EMT and metastasis by repressing HDAC3 expression (Ma et al. 2020). These findings present a regulatory network that involves many key players in the EMT program.

Honokiol is a small-molecule polyphenol attained from the bark of the genus Magnolia. It has potential anti-tumor activity in various cancer models (Fried and Arbiser 2009). Intravenous injection of 5–10 mg/kg Honokiol in experimental rats reveals that its half-lives were 49.22 ± 6.68 or 56.24 ± 7.30 min (Chiu et al. 2019; Fried and Arbiser 2009). Meanwhile, results of intra-peritoneal injection following 250 mg/kg can yield a *t*_1/2_ of 4–6 h, with a *t*_max_ of about 20–30 min in mice. Honokiol retention time is 4.94 min (Liu et al. 2015). Honokiol has unusual structural motifs among phenolic antioxidants and these motifs feature isomeric bisphenolic cores bearing allyl substituents (Amorati et al. 2015). Our previous serial studies have revealed that Honokiol can induce cancer cell apoptosis and inhibit tumorigenesis and metastasis of gastric cancer cells via endoplasmic reticulum (ER) stress-related signaling pathways and specific targeting of key calpain activation, as well as the downregulation of CRT, PPARγ, Grp94, COX-2, STAT-3, and Tpl-2 proteins (Liu et al. 2015, 2012; Pan et al. 2013a; Sheu et al. 2007). A recent study has also shown that Honokiol can inhibit EMT in the breast cancer cells through downregulation of Snail/Slug protein translation (Wang et al. 2019). However, the anti-EMT potential of Honokiol and its correlation with HDAC3 regulation have not been investigated in gastric cancer in vitro and in vivo.

The present study aimed to clarify the role of Honokiol in suppressing HDAC3 activation on EMT and metastasis in gastric cancer. This study is for the first time to elucidate the role of Honokiol-induced changes in HDAC3 (Y298) site and C/EBPβ, NFκB, on EMT and on peritoneal dissemination in gastric cancer. The results reveal that the invasiveness of gastric cancer cells, largely dependent on the over-expression of the C/EBPβ/NFκB/HDAC3 signaling cascade, is indispensable for the invasiveness of pathologic tissues. Such findings provide extremely valuable information regarding the target genes in extracted clinical tissue that may be used for survival analysis. The results from clinical tissues analysis reveal that HDAC3 overexpression is associated with tumor recurrence and poor prognosis. Furthermore, our in vivo and in vitro studies, which tracked gastric cancer cells in a mouse model of peritoneal carcinomatosis, demonstrates for the first time how Honokiol therapy, pharmacological inhibition, and gene silencing of HDAC3 effectively suppress the parietal peritoneum, thereby eliminating metastatic cancer cells. We also found that the HDAC3 knockdown in cancer cells inhibits EMT markers and activates ER stress in vivo.

## Material and methods

Most of the methods used here have been described in our previous studies (Chiu et al. 2019; Lee et al. 2013; Liu et al. 2015, 2010, 2012; Pan et al. 2013a, b; Wu et al. 2016b). Honokiol was obtained from the Wako Chemical Company (Osaka, Japan).

### Cell culture

Cell culture systems were performed as previously described (Wu et al. 2016b). The cell bank of Taipei Veterans General Hospital (Taiwan) supplied the human gastric cancer cell lines, AGS (moderately differentiated gastric adenocarcinoma) and MKN 45 or SCM-1 cells (poorly differentiated gastric adenocarcinoma). Cells were grown in RPMI medium supplemented with 10% FBS, 100 U/ml penicillin, and 100 mg/ml streptomycin (complete medium) at 37 °C in a humidified incubator with 5% CO_2_. During experiments, cells were plated in six-well plates cultured with serum-free medium (starved medium) overnight and then treated with drugs (honokiol or melatonin) with various concentrations for various time intervals. In some experiments, transfection of cancer cells was performed using a Lipofectin reagent (Invitrogen) according to the manufacturer’s instructions. The efficiency of transfection (~ 90%) was determined using an equal amount of a plasmid that encoded the green fluorescent protein under the cytomegalovirus promoter.

### Tissue samples and clinical data collection

Tissue samples from 40 patients with gastric cancer were analyzed. The institutional review board of Taichung Veterans General Hospital approved the study protocol (Approval No CE 17096B). Data obtained from the registry of the Cancer Institute of Tissue Bank included age, sex, differentiation, tumor location, tumor size, depth of tumor invasion (T stage), and number of metastatic lymph nodes (N stage) (both based on the seventh edition UICC TNM classification for gastric cancer), extent of lymph node metastasis, and Lauren classification. Correlations between HDAC3 expression and the clinicopathologic characteristics of patients were summarized. Fisher’s exact test was used for the histologic analysis of tumor stage, tumor grade, and distant metastasis, while one-way analysis of variance (ANOVA) was used to compare clinicopathologic characteristics. Statistical significance was set at *p* < 0.05.

### Ligand binding assay

This assay was conducted as previously described in 10 mM 2-(N-morpholino) ethanesulfonic acid (MES) buffer containing10 mM MnCl2, 1 mM EDTA, adjusted to pH 6.0 with KOH (Davis and Sharif 2000). Initial tissue linearity studies were carried out using 0.1 ± 200 μg of the cell containing the recombinant human HDAC3 per 0.5 ml total volume in 96-well deep-well assay blocks (Matrix Technologies Corp., Hudson, NH, USA), using 300 pM [3H]-Honokiol (PerkinElmer Inc.). Proteins were thawed quickly, diluted to the desired concentration in the binding buffer, and mixed to a homogeneous suspension prior to dispensation. After the addition of the radioligand, the assay mixtures were incubated for 120 min on a rotary shaker (50 rpm). The reaction was terminated by rapid vacuum filtration on Whatman GF/B glass filter mats (previously soaked in 0.5% polyethyleneimine) using cold MES buffer. Bound radioligand was then quantitated by liquid scintillation counting (Packard BioScience).

### Differential scanning calorimetry

Differential scanning calorimetry (DSC) analysis was performed as described previously by Martin et al. (2013). DSC measurement was determined by a PerkinElmer DSC 7 Differential Scanning Calorimeter (Waltham, MA, USA). Although some HDAC3 molecules may have nucleotide bound at the end of the purification, this will be released from the protein before the protein unfolding.

### Immuno-histochemistry

In the human gastric cancer tissues, 5-μm-thick sections were cut from 10% formalin-fixed samples and stained for specific antibodies using anti-primary antibodies. Immuno-histochemistry (IHC) analysis was performed as described previously (Liu et al. 2015). All antibodies used were listed and described in Supplementary Tables 1 and 2.

### HDAC3 activity

A HDAC3 Activity Assay Kit (Abnova Corporation; Catalog Number KA3731) was used to examine HDAC3 activity. A quantitative fluorometric determination of HDAC3 activity was measured according to the manufacturer’s protocol. The assay kit provided a positive control (a HeLa nuclear extract), a deacetylated HDAC assay standard, and a control inhibitor (trichostatin A; TSA), as well as a colorimetric HDAC3 substrate [R-H–K-K(Ac)-AFC] to release the AFC molecule, which could be fluorometrically detected (Ex/Em = 380/500 nm).

### Luciferase reporter assay

Genetic reporters were used as indicators of gene expression studies with accompanying cellular events (Pan et al. 2013a). Cells at 60% confluence were co-transfected with 0.2 μg of the promoter-reporter construct CCAAT/enhancer-binding protein B (C/EBPβ), NFκB activator, and 0.05 μg of a thymidine kinase promoter-driven Renilla luciferase vector (pRLTK; Promega, Mannheim, Germany). The pRL-tk-LUC vector coding for a Renilla luciferase under the control of a constitutively active thymidine kinase promoter was co-transfected to correct for transfection efficiency. After incubation, cells were lysed and processed using the Dual-Luciferase Kit (Promega) as described by the manufacturer. Luciferase activity was normalized against the Renilla firefly activity for transfection efficiency and recorded by a luminometer (LKB, Rockville, MD, USA). Experiments were performed in triplicate, unless stated otherwise.

### Endoplasmic reticulum stress response element

Endoplasmic reticulum stress response element (ERSE) measurement was performed using a Cignal ERSE Reporter Luciferase assay kit (SA Biosciences, QIAGEN, Frederick, MD, USA). This assay kit contained a mixture of an ERSE-responsive luciferase construct and a constitutively expressed Renilla luciferase construct. The diluted transfection mixtures provided ready reporter, negative control, positive control formulations, and relevant nucleic acid tests. Overnight post-transfection, the transfected cells were treated with Honokiol and ER stress activator. Activities of the signaling pathways were investigated using dual luciferase assay.

### Transmission electron microscopy

Transmission electron microscopy (TEM) was performed as previously described (Pan et al. 2013b). Cells were treated with or without Honokiol or shHDAC3 for 18 h and then harvested and fixed with 4% glutaraldehyde and 2.5% paraformaldehyde dissolved in 0.1 M sodium cacodylate. Cells were then post-fixed in 1% osmium tetraoxide, dehydrated in ethanol, and embedded in araldite. Sections on grids were counter-stained with uranyl acetate and lead citrate, and examined in a JEM 1200 EX TEM (JEOL, Peabody, MA, USA) at an accelerating voltage of 80 kV.

### Transfection

Cancer cells AGS, SCM1, and MKN45 were transfected with shRNA (National RNAi Core Facility Platform, Taipei, Taiwan) or overexpressed plasmid 1 μg/ml pcDNA (Genome Research Center, National Yang-Ming University) using a Lipofectin reagent (Invitrogen), in accordance with the manufacturer’s instructions.

### Immuno*-*blotting

The preparation of whole-cell lysates of gastric cancer cells for immuno-blotting was performed as previously described (e.g., Liu et al. 2015; Pan et al. 2013a). All antibodies used in the present study were also listed in Supplementary Tables 1 and 2. Detection was performed by ECL (Amersham) and by chemiluminescence using Kodak X-Omat film. The immunoblot assay was independently repeated five times.

### Wound-healing cell migration assay and Matrigel invasion assay

After transfection, cells were seeded at 3.5 × 10^5^ cells/well in 12-well plates. At 100% confluence, cells were scraped by a sterile tip of a 1000 μl pipette to generate an artificial “wound.” Two parallel wounds were created using a plastic pipette tip. Cells were further grown in a culture medium with 2% FBS. The images were collected at 0 and 18 h using a microscope. Migration capacity was quantified by measuring the change of “wound” width. This assay was independently repeated five times.

For the Matrigel invasion assay, 3 × 10^5^ cells/well were seeded in the upper chamber that was coated with Matrigel (BD Bioscience, San Diego, CA, USA). After 48 h at 37 °C and 5% CO_2_, cells present on the lower surface of the insert were stained with Diff-Quik stain (Biochemical Sciences, Inc., Swedesboro, NJ, USA). Cells that invaded through the Matrigel-coated membrane were microscopically enumerated. Cell staining from three randomly selected fields was photographed using a CKX41 inverted microscope (Olympus Corp). The mean value was recorded. All experiments were performed in triplicate; each being repeated at least five times. And this variability of the mean values is represented by the SD.

### Experimental animals

All animal studies were approved by the appropriate institutional ethical committee of the Taichung Veterans General Hospital of Taiwan (Approval No La-1061488). All procedures have been conformed according to the guidelines from the NIH Guide for the Care and Use of Laboratory Animals. All animals were euthanized by cervical dislocation under isoflurane inhalation (induction: 3%, maintenance: 1–2%) in medical air (0.4 L/min).

### Xenograft tumor mouse model and positron emission tomography-computed tomography

Preparation of cancer cells was conducted as described previously (Chiu et al. 2019). Imaging studies were performed using positron emission tomography-computed tomography (PET/CT). To evaluate the peritoneal metastasis, MKN45 cells were inoculated into the peritoneal cavity of BALB/c nude mice. Peritoneal tumors in the nude mice were established by PET/CT surveillance 5–7 days after the injection. Mice were administered with Honokiol (5 mg/kg/twice per week) for 28 days by intra-peritoneal injection.

Changes in the peritoneal dissemination by Honokiol administration were evaluated by PET/CT. Mice were euthanized under anesthesia and examined macroscopically for the presence of peritoneal metastasis. The tumors were excised, cut into blocks, fixed in 10% formalin, and embedded in paraffin blocks or snap-frozen in liquid nitrogen. All images with all available clinical information were analyzed and interpreted by the senior nuclear medicine physicians. Correlatively conventional imaging was used for anatomic guidance.

### TOP/FOP luciferase reporter assay

To assess the transcriptional activity of β-catenin in gastric cancer cells, the TOP/FOP reporter system using the dual-luciferase kit (Dual-Glo™ Luciferase Assay System, Promega, Madison, WI, USA) was used. Cells were transiently transfected with 1 μg of the constitutively active vector encoding thymidine kinase promoter-Renilla luciferase reporter plasmid (pRL-TK) (Promega) and β-catenin responsive firefly luciferase reporter plasmid TopFlash (Millipore, Billerica, MA, USA), or the negative control FopFlash (Millipore) using lipofectin. Cells were harvested after 24 h in culture and both firefly and Renilla luciferase activities were measured in duplicate/triplicate according to the manufacturer’s instructions. The firefly luciferase activity was normalized against the Renilla luciferase activity and the fold increase in TOPFlash activity compared to FOPFlash was reported.

To assess the function of Honokiol on β-catenin transcriptional activity, cells were co-transfected with TopFlash or FopFlash and small hairpin RNA (shRNA) using lipofectin. Cells were harvested after 36 h for luciferase measurements. Luminescence was read using a Sirius luminometer (Berthold Detection System, Pforzheim, Germany).

### Statistical analyses

Values were presented as mean ± SD. Analysis of variance (ANOVA), followed by Fisher’s least significant difference test, was performed for all data. Statistical significance was set at *p* < 0.05.

## Results

### High HDAC3 expression levels decreased overall survival probability in gastric cancer patients

**Fig. 1:**
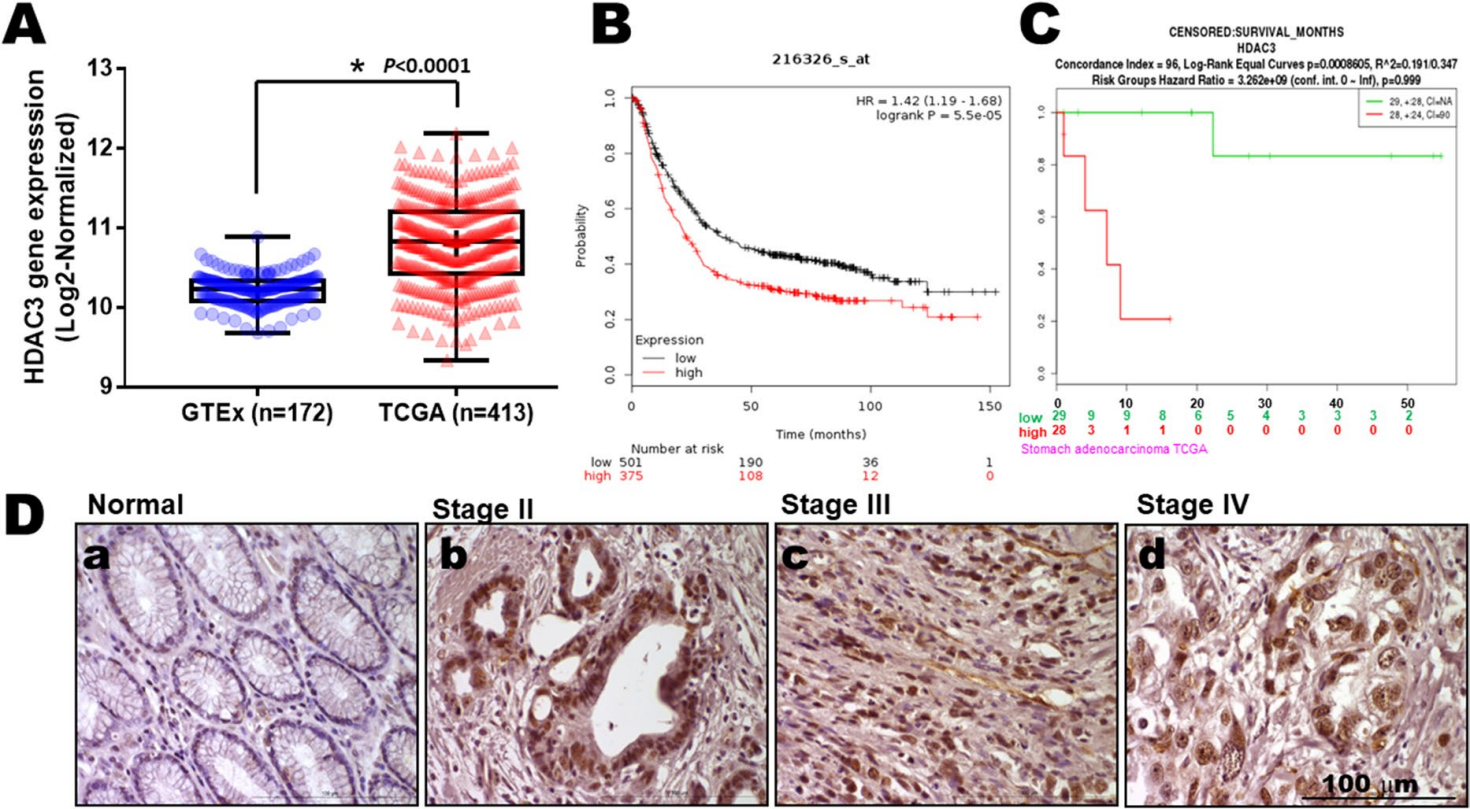
High HDAC3 expression in patients with gastric cancer correlated with survival rate. **A** HDAC3 expression levels in normal tissues and tumor samples derived from publicly available Genotype-Tissue Expression (GTEx, *n* = 172) and The Cancer Genome Atlas (TCGA, *n* = 413) gene expression data, respectively, plotted as box and whisker plots. OS stratified by quartiles distribution.** B** High HDAC3 expressions were associated with decreased overall survival probability in gastric cancer. Data obtained were from the dataset (216326_s_at, *n* = 876) through a comprehensive search using Kaplan–Meier plotter.com for HDAC3 evaluation. **C** Enhancements of the existing database SurvExpress for HDAC3 (*n* = 57). **D** HDAC3 is upregulated in gastric cancer tissues from the Taichung Veterans General Hospital (TCVGH) Tissues Bank (*n* = 40). (a) Representative immuno-staining of HDAC3 expressions in human normal gastric mucosa. (b) Poorly differentiated intestinal adenocarcinoma, (c) diffused adenocarcinoma in mucosa, and (d) diffuse adenocarcinoma in serosa are shown. Scale bar = 100 μm

Data from the Genotype-Tissue Expression project and the Cancer Genome Atlas were first integrated to comprehensively analyze the transcriptomes of 172 healthy and 413 tumor tissues (Fig. 1A). By web-based correlation, there was a markedly positive correlation between the high expression levels of HDAC3 in 876 patients with gastric cancer, using the selected parameters and run on by Kaplan–Meier plotter (KMplot.com), Probability GSE216326 dataset, or by TCGA (Fig. 1B). The desired Affymetrix ID is valid: 216326_at **(**HDAC3). Survival curves were plotted for all patients with gastric cancer (*n* = 876) using the Kaplan–Meier analysis, with HR of 1.42 (1.19–1.68) and logrank *p* = 5.5e-5. In addition, similar patterns were also found in SurvExpress Web resource in stomach cancer of the TCGA dataset (*n* = 57) (Fig. 1C).

Immuno-histochemical analysis of human gastric cancer specimens demonstrated an increase in HDAC3 expression compared to benign tissues adjacent to the tumor (Fig. 1D-a). A survey of the benign tissue revealed mostly typical moderately differentiated adenocarcinoma (Fig. 1D-b). In diffused-type gastric cancer tissues (Fig. 1D-c), adenocarcinoma with lymph node and distant metastasis was the dominant finding (Fig. 1D-d). The percentage of positive tumor cells and staining intensity for each sample were recorded. These results indicated that the level of HDAC3 expression closely correlated with increased clinical stage, as well as with lymph node and distant metastasis of the tumor-node-metastasis (TNM) classification.

### Pharmacological characterization of [^3^H]-Honokiol binding to HDAC3

**Fig. 2:**
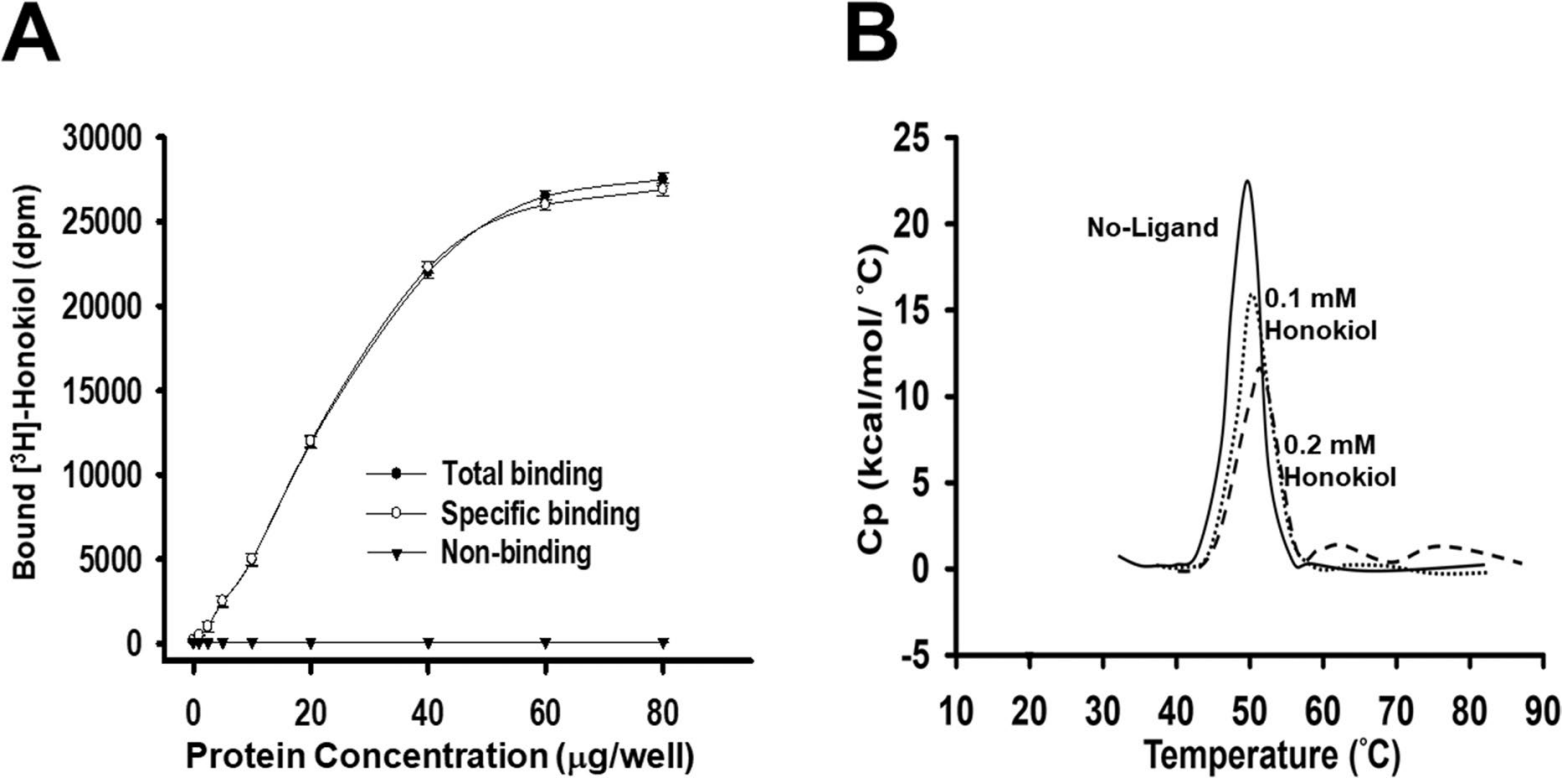
Pharmacological characterization of [3H]-Honokiol binding to HDAC3. **A** Biological tissue linearity of [^3^H]-Honokiol binding to HEK-293 cell membranes expressing the recombinant human HDAC3. Samples were added in varying amounts to 0.2 nM [^3^H]-Honokiol in a final volume of 500 μl and the binding assay was performed as described in the “Materials and methods” section. Data from a representative experiment are shown. Note the very high level of specific binding found in this system. All the results were expressed at least three independent experiments and presented. **B** The purpose of this protocol is to prepare HDAC3 (10 μM) in 50 mM HEPES, 0.1 M KCl and 10 mM beta-mercaptoethanol pH 7.2 was contingent thermal denaturation in the presence or absence of Honokiol for DSC. Subsequently, samples were heated from 25–80 °C at a rate of temperature of 90 °C per hour. Solid lines, no ligand; dashed lines, 0.1 mM Honokiol; dotted lines and dashed 0.2 mM Honokiol. The contours were essentially the same when determined in a buffer consisting of 50 mM potassium phosphate, pH 7.2, 1 mM dithiothreitol. All the results were expressed at least six independent experiments and presented

The basis on unpublished observations in microarray analysis screen, we found Honokiol markedly reduced HDAC3 expression, but not HDAC1/2. In the study, we targeted HDAC3 research. To investigate whether Honokiol targets HDAC3, we used the assays of binding affinity and DSC to verify its pharmacological characterization. The binding of [^3^H]-Honokiol to HEK-293 cell membranes, which expressed the recombinant human HDAC3, was depicted in Fig. 2A. Specific ligand binding assay was determined. Honokiol efficiently targeted on HDAC3, but limited on HDAC1 and HDAC2 (Supplementary Fig. 1A). Moreover, DSC analysis showed the shifts of protein melting temperature (Tm) that occurred upon ligand binding to a protein giving a stabilized complex. To characterize the binding of Honokiol to HDAC3, DSC analysis was used to unfold HDAC3 in the absence and presence of Honokiol (0.1 mM and 0.2 mM). As shown in Fig. 2B, Honokiol bound with the greatest affinity to the nucleotide-binding domain (NBD) as decided by the relative changes in the melting temperature (Tm). A shift of the denaturation peak to higher temperatures in the presence of a ligand is a sign of protein–ligand binding. The changes in Tm implied that Honokiol was bound to the unfolded form of HDAC3. A typical thermogram of unliganded HDAC3 was also shown in Fig. 2B. These findings from the ligand–protein binding specificity study proved the direct and convincing evidence that Honokiol could directly interact with HDAC3.

### Honokiol inhibited the activity and Tyr (Y298) phosphorylation of HDAC3 in gastric cancer cells

**Fig. 3:**
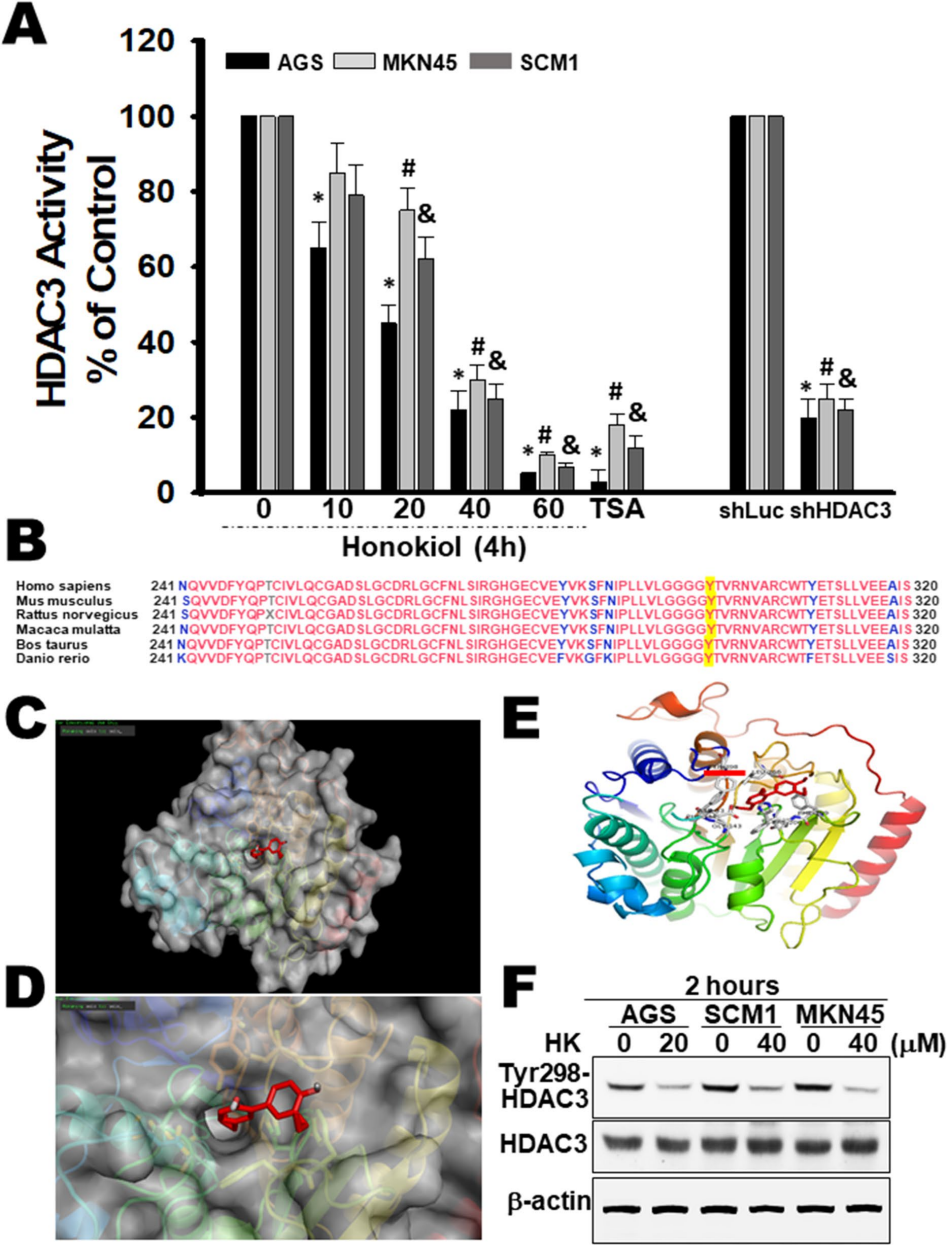
Honokiol inhibited HDAC3 enzymatic activity in gastric cancer cells. The chemical structure of Honokiol revealed binding modes in HDAC3 by molecular docking simulation. General and local overviews of the best interaction after automated docking of binding Honokiol (red) to the active site of HDAC3 were shown. **A** Exposure to Honokiol inhibits HDAC3 enzymatic activity in gastric cancer cells AGS, SCM1, and MKN45 in a dose-dependent manner. Trichostatin A (TSA) and gene silencing by shHDAC3 (5 μg/ml) served as a positive control. HDAC3 activity was measured by colorimetric HDAC3 Activity Assay Kit. The results are representative of at least six independent experiments and presented as mean ± SD; *p < 0.01 versus untreated cells **B** Alignment of the sequences of the HDAC3. Sequences are the amino acids for residues 241–320 of the proteins. Organisms were Homo sapiens, Homo sapiens, Mus musculus, Rattus norvegicus, Macaca mulatta, Bos Taurus, Danio rerio. Sequences are from Genebank. **C** Surface representation of the ligand-receptor binding. Honokiol and HDAC3 are shown in surface representation. **D** Local interaction positions of the HDAC active site. The cave entrance of zoom-in binding mode of HDAC3 and Honokiol is shown. **E** The molecular structure of human HDAC3 with Tyr 298 is depicted as a ribbon diagram, with α-helices, β-pleated sheets, and loops. The binding mode of HDAC3 and Honokiol is shown targeting Tyr 298 representation. The 3D representation of the interactions of Honokiol binding to the HDAC3 active site, as generated by PyMol. **F** Honokiol-treated cancer cells (AGS 20 μM; SCM1 40 μM; MKN45 40 μM) markedly reduced the specific antibody targeting Tyr (Y298) of HDAC3 at 2 h, whole-cell lysates as evaluated by western blotting. The results are representative of at least six independent experiments and presented

To determine the effects of Honokiol on HDAC3 activities in vitro, AGS and SCM1 cells were treated with Honokiol (10–60 μM) or with Trichostatin A (TSA; an inhibitor of HDAC) for 4 h. Honokiol treatment showed significant inhibition of HDAC3 activity in a dose-dependent manner compared to vehicle control (Fig. 3A). Gene silencing of shHDAC3 presented a similarly inhibitory pattern. In addition, we also determined the effects of Honokiol on the HDAC1/2 activities, but limited effects on it (Supplementary Fig. 1B-C).

We next investigated the evolutionary sequence conservation using orthologous proteins. Sequence alignment was the amino acids for residues 241–320 of the HDAC3 proteins (Fig. 3B). Results showed that key residues within the HDAC3 catalytic site interacted with Honokiol and bound in the active site tunnel (Fig. 3C and D). Crystal structures of HDACs revealed that the highly conserved Tyr residue (Y298 in HDAC3) was located within the active site (Fig. 3E) in which was catalytically required in stabilizing the molecular functional activity. Changes in both conformation and dynamics might occur when Honokiol was bound to HDAC3, facilitating Honokiol targeting to the active site. This was catalytically essential in stabilizing the tetrahedral intermediate and polarizing the substrate carbonyl group (Amorati et al. 2015), resulting in the blockage of HDAC3 activity (Lombardi et al. 2011; Sun et al. 2013; Watson et al. 2012).

Furthermore, specific antibody targeting Tyr (Y298) phosphorylation of HDAC3 in three gastric cancer cell lines was markedly reduced by Honokiol after 2 h treatment (early stage) (Fig. 3F), but not constitutive HDAC3 per se. We also assessed the protein levels of HDAC3 at 12–24 h. And results revealed that treatment of cells with Honokiol resulted in a reduction in the levels of the HDAC3 compared to the control cells. We also simultaneously examined the mRNA and protein expression levels in HDAC1 and HDAC2, but no changes were observed (Supplementary Fig. 1D-E).

Taken together, these results indicated the possible interaction between HDAC3 and Honokiol via structure molecular docking as well as HDAC3 kinase functional activity. Based on the above mentioned in Fig. 2 and Fig. 3, evidence identifies that Honokiol is a direct target of HDAC3 by the post-translation modification and gene regulation.

### Honokiol-induced ER stress inhibited the activities of NFκB and CEBP/β to restrain the HDAC3 activation in gastric cancer cells

**Fig. 4:**
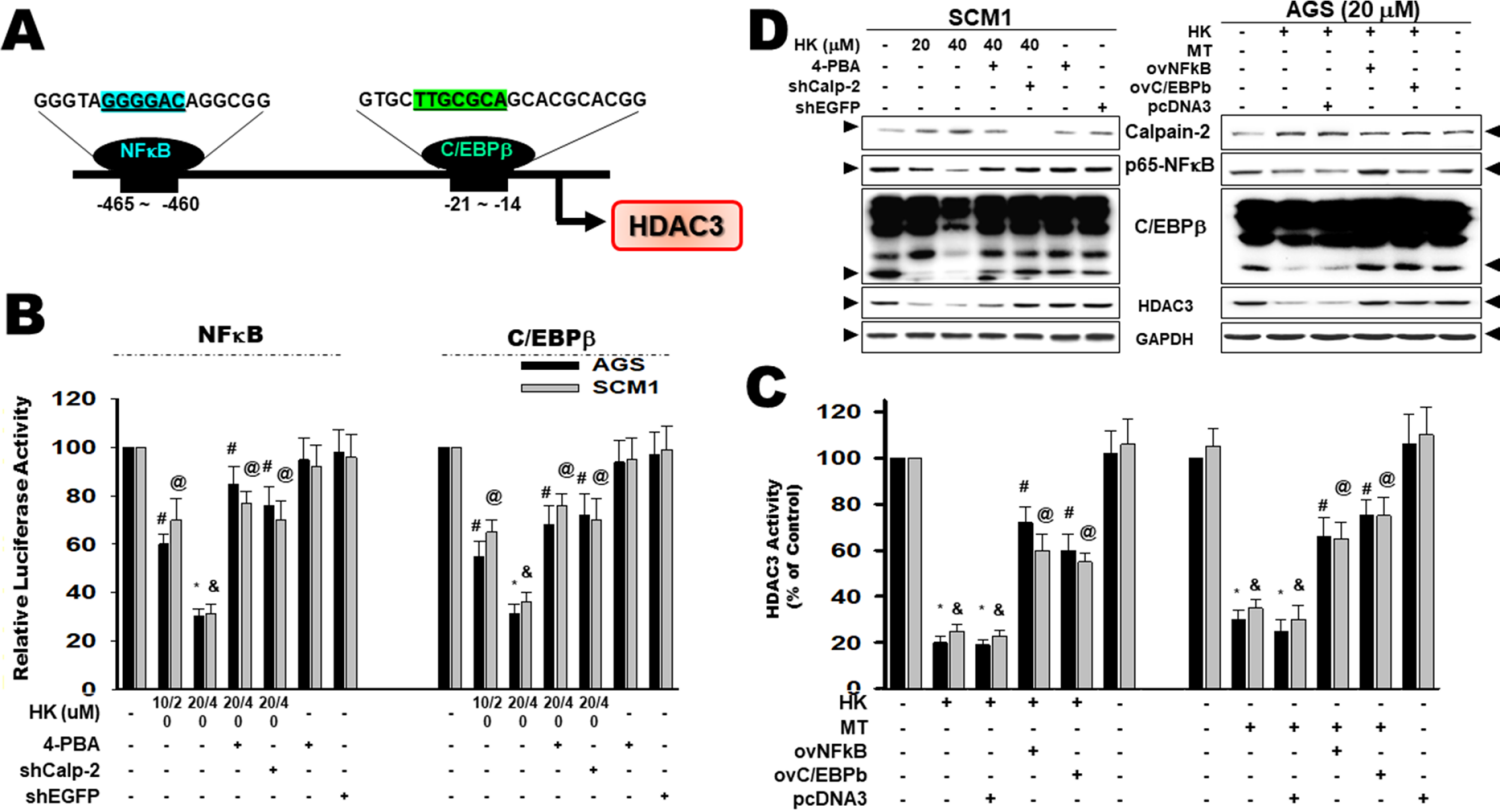
Honokiol suppressed HDAC3-regulated NFκB and C/EBPβ signaling in gastric cancer cells. **A** The sequences of NFκB and C/EBPβ in HDAC3 promoter are presented, indicating the sub-fragments used in this study and the consensus minimal promoter (underlined as indicated). Promoter regulation in the HDAC3 promoter-flanking region (− 465 ~  − 460) contained the cis-acting elements NFκB DNA binding site. HDAC3 promoter-flanking region (− 21 ~  − 14) contained the transcription repression C/EBPβ DNA binding site. **B** Honokiol reduced the activation of C/EBPβ (right) and NF-κB (left). The relative activity was measured by dual-luciferase activity kit as described in the “Materials and methods” section. All data are presented as mean ± SD of six separate determinations. ^#@^*p* < 0.05 and *^&^*p* < 0.01 versus control cells. **C** SCM1 or AGS gastric cancer cells were treated with Honokiol (40 μM) or melatonin (MT; 100 mM) for 24 h, and transiently transfected with NFκB plasmid or C/EBPβ plasmid for HDAC3 activity. This was determined in a test sample. All data are presented as mean ± SD of six separate determinations. ^#@^*p* < 0.05 and *^&^*p* < 0.01 versus control cells. **D** Gene silencing or overexpressed plasmid as indicated in SCM1 gastric cancer cells were treated with Honokiol (40 μM) for 24 h. Whole-cell lysates as evaluated by Western blotting. The results are representative of at least three independent experiments and presented

HDAC3 includes numerous transcription factors such as NF-κB and C/EBPβ binding sites (Paz-Priel et al. 2011; Somech et al. 2005). Accordingly, promoter regulation in the NF-κB and C/EBPβ promoter-flanking region (− 465 ~  − 460 and − 21 ~  − 14, individually) containing the cis-acting elements DNA binding activity in silico was also hypothesized (Fig. 4A). A previous study showed that Honokiol induced ER stress cascade and activated Calpain-II activity and protein expression (Liu et al. 2012). To determine if Honokiol-induced ER stress inhibited NF-κB and C/EBPβ signals, the transcriptional activity was measured using a luciferase reporter assay (Fig. 4B). Exposure to Honokiol effectively prevented the activities of NF-κB (Fig. 4B, left panel) and C/EBPβ (Fig. 4B, right panel), which could be reversed by 4-phenylbutyric acid (4-PBA; a chemical chaperone) and gene silencing of Calpain-2 (shCalp-2). Results showed that both NF-κB and C/EBPβ were downstream-regulated by Honokiol treatment and this was associated with ER stress induction. These results suggest that ER stress plays an important role in the regulation of transcriptional activities of transcription factor NF-κB and C/EBPβ axis in gastric cancer cells in response to Honokiol.

In addition, Honokiol treatment decreased the HDAC3 activity in AGS or SCM1 cells, which could be reversed by the over-expression of C/EBPβ (ov-C/EBPβ) and NF-κB (ov-NF-κB) transfection compared to those in control pcDNA3 cells (Fig. 4C, left panel). Positive control by Melatonin (Wu et al. 2016a, b) treatment in both ov-C/EBPβ and ov-NF-κB-transfected cells compared to control cells was also statistically confirmed (Fig. 4C, right panel). The expression of proteins for Calpain-2, p65-NF-κB, C/EBPβ, and HDAC3 was shown in Fig. 4D. These findings indicated that Honokiol reduced NF-κB and C/EBPβ activation, resulting in the down-regulation of HDAC3 activity. This may have potential in the development of treatment strategies for the peritoneal dissemination of gastric cancer.

### High expression of NF-κB-p65, C/EBPβ, and HDAC3 decreased overall survival probability in gastric cancer

**Fig. 5:**
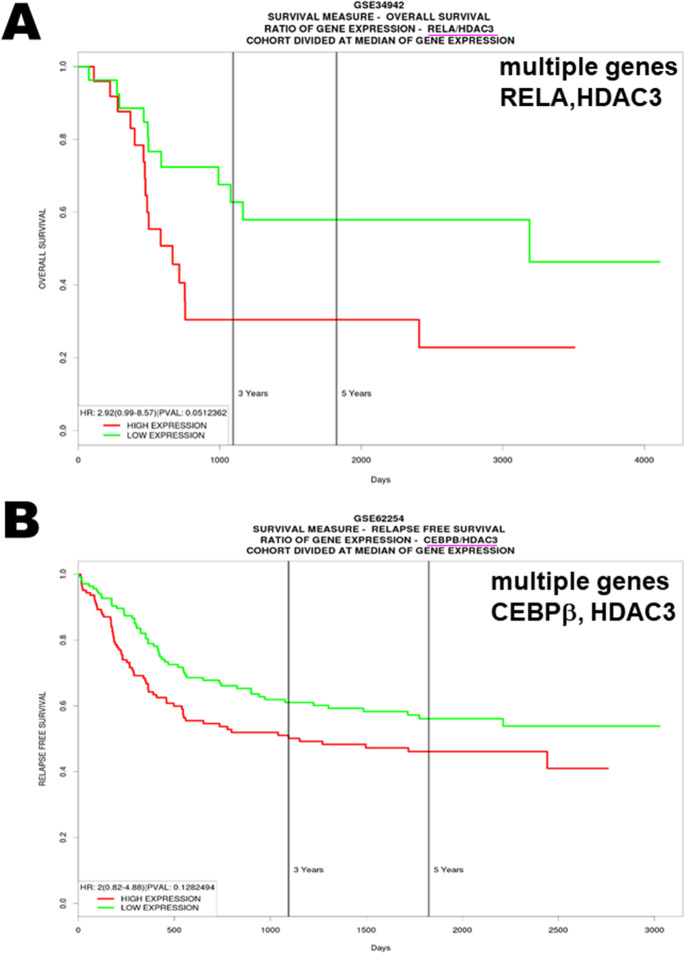
High levels of NFκB-p65 (RELA), C/EBPβ, and HDAC3 expression were associated with decreased overall survival probability in gastric cancer. **A** Data were from the Survival dataset (GSE34942) through a comprehensive search using SurvExpress for RELA and HDAC3 evaluation. **B** Enhancements of the existing database (GSE62254) PROGgeneV2 for C/EBPβ and HDAC3

Using web-based applications to conduct KM plot, univariate hazard ratio, landmark analysis, quantile survival analysis, and competing risk analysis, there were significant positive correlations between the high expression of NF-κB-p65 (Rel A), C/EBPβ, and HDAC3 in gastric cancer and overall survival and relapse-free survival, as annotated in the GSE34942 for NF-κB-p65 (RELA) and GSE62254 for C/EBPB datasets by TCGA as presented in the PROGgeneV2 (Fig. 5A and B, respectively). Both NF-κB and C/EBPβ were also confirmed in patterns found in 876 patients with gastric cancer represented in the KM plot software. The public database and web application KM plotter (Supplementary Fig. 2A and B, respectively) and the SurvExpress Web resource created the Kaplan–Meier curves based on the NF-κB-p65 (RELA), C/EBPB, and HDAC3 expression levels (Supplementary Fig. 3). Taken together, these findings suggested that NF-κB-p65, C/EBPβ, and HDAC3 were constitutively activated in human metastatic gastric adenocarcinoma. Pharmacological reduction of NF-κB-p65, C/EBPβ, and HDAC3 expression might be useful surrogate markers for treatment activity.

### Honokiol-induced ER stress in gastric cancer cells

**Fig. 6:**
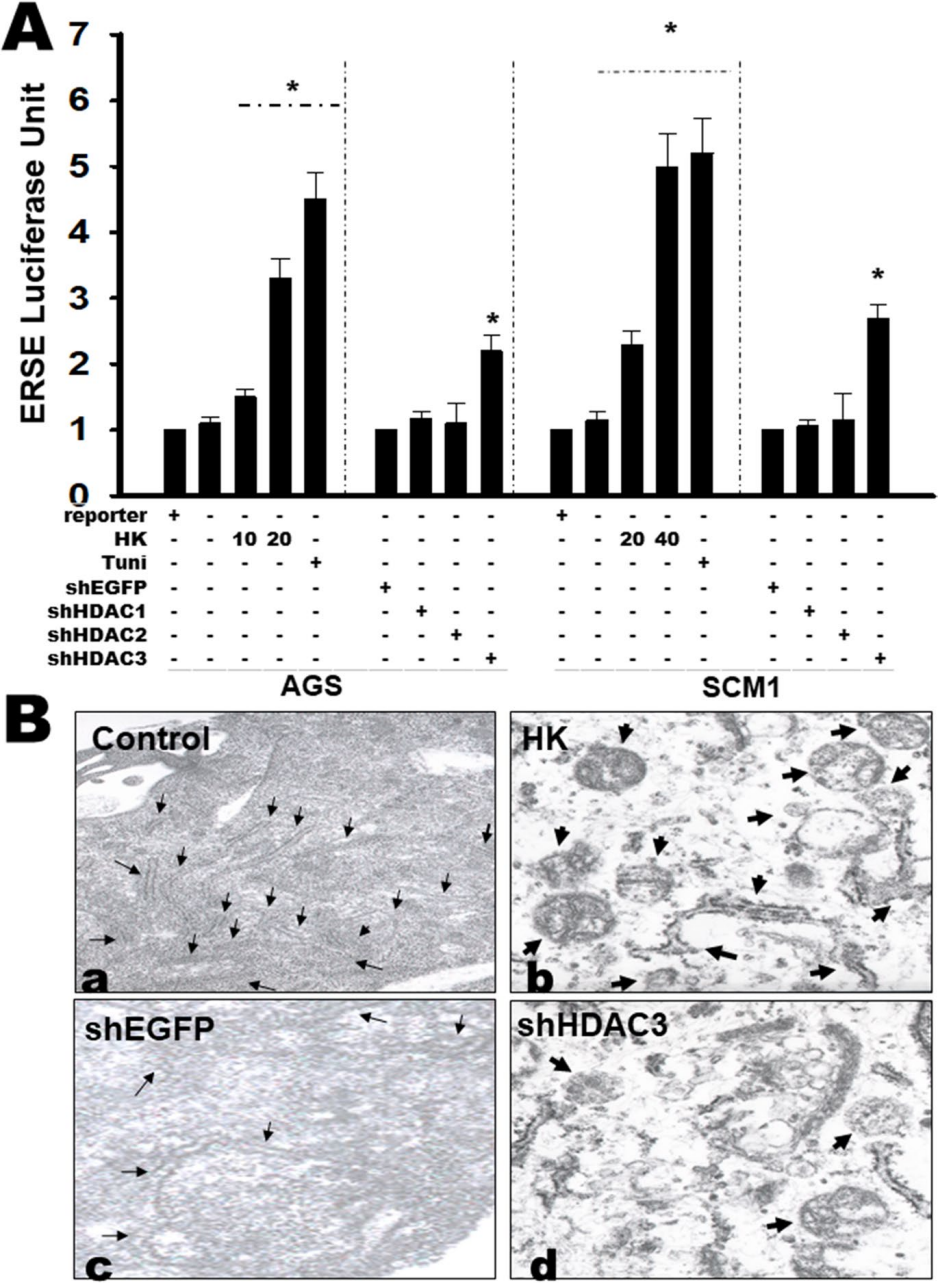
Honokiol and gene silencing of HDAC3 induced ER stress in gastric cancer cells. **A** The Cignal TCF/LEF reporter assay was designed to monitor the activity of Wnt signal transduction pathways in cultured AGS or SCM1 cells after transfection with shEGFP or shHDAC3 and then exposed to Honokiol after 18 h. The data are representative of at least six independent experiments. Data are presented as mean ± SD (*n* = 6). ERSE reporter contained inducible transcription-factor-responsive GFP reporter; Negative control included GFP reporter construct with GFP expression controlled by a minimal promoter; Positive control consist of constitutively expressing GFP construct. ER stress inducer Tunicamycin 0.1 μg/ml was the positive control. All data are presented as mean ± SD of six separate determinations. *p < 0.05 versus control cells. **B** ER stress was examined by transmission electron microscopy. Cells were collected and visualized by electron microscopy as described in the “Materials and methods” section. The results shown are representative of typical experiments. (a) Control AGS cells; (b) Honokiol-treated cell displays ER dilation; (c) cell transfection shEGFP alone; and (d) cell transfection shHDAC3 alone induces serious ER dilation with increased distention and a fragmented organelle. Arrows indicate dilated ER; Original magnifications, 9800 K

Exposure of gastric cancer cells AGS or SCM-1 to Honokiol or Tunicamycin (positive control; an ER stress inducer) led to a marked 2.3- to 5.8-fold increase of ERSE luciferase activity compared to control cells (Fig. 6A). Gene silencing of HDAC1/2/3 also induced ERSE activity. The effects of shHDAC1/2 were very weak or limited. These cells were identified unequivocally as evidence of ER stress induction, which was further confirmed by qualitative ultrastructure analysis using a transmission electron microscopy (TEM) analysis for cell organelle structure morphology. In control cells, the abundant strands of rough ER with narrow cisterns were surveyed (Fig. 6B-a, arrows). However, after 12-h Honokiol treatment, a large part of the cisterns showed dilated organelles and was swollen into circular shapes (Fig. 6B-b, arrows). There was an unexpected finding in the HDAC3 gene silencing (shHDAC3) group (Fig. 6B-d) presenting with a similar contour of dilated organelles in ER imaging compared to the control (shEGFP) group (Fig. 6B-c). These results are correlated with previous findings of network regulation and control of ER stress by Honokiol exposure.

### Honokiol inhibited EMT marker expression and cell invasion via NF-κB-p65 and C/EBPβ inhibition in gastric cancer cells

**Fig. 7:**
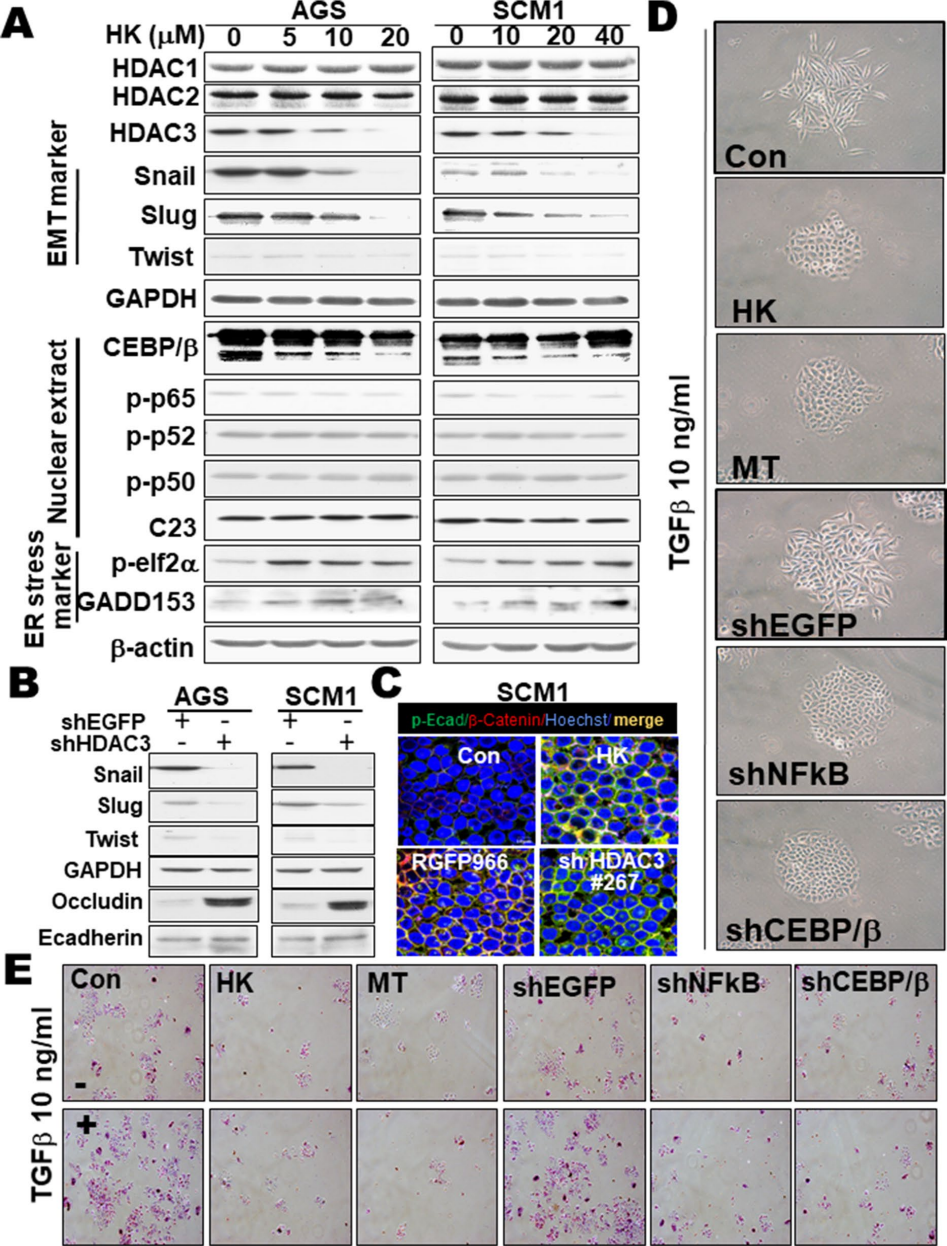
Honokiol and gene silencing of HDAC3 attenuated the EMT markers, cell invasion, and sphere formation ability of gastric cancer cells. **A** Honokiol (HK) had a dose-dependent effect on cells, as detected by western blotting. The EMT markers (Snail, Slug, Twist), transcription factor (C/EBPβ, NFκB p-p65, p-p52, and p50), ER stress markers (p-elf2α, GADD153) were all evaluated. The images are representative of at least five independent experiments. **B** Knockdown HDAC3 by gene silencing of HDAC3 (shHDAC3#267) suppressed EMT markers and increased Occludin, but not E-cadherin or p-E-cadherin (data not shown). **C** Cells were exposed to Honokiol, HDAC3 inhibitor RGFP966, and gene silencing of HDAC3 (shHDAC3#267) in a dose-dependent manner and then fixed and incubated with monoclonal antibodies against specific p-E-cadherin (green), β-Catenin (red) and Hoechst (blue). Confocal images for p-E-cadherin, β-Catenin, and Hoechst were examined and quantified as described in the “Materials and methods” section. The images are representative of at least five independent experiments. **D** SCM1 cells were treated with TGFβ (10 ng/ml) in the presence or absence of Honokiol (HK), melatonin (MT), or transfection with expressing shEGFP (control), shNF-κB, or shCEBPB and subsequently seeded onto 24-well plates for one week for morphological examination. The images are representative of at least five independent experiments. **E** For invasion assay, SCM1 cells were treated with TGFβ (10 ng/ml) in the presence or absence of Honokiol (HK), melatonin (MT), or transfection with expressing shEGFP (control), shNFκB, or shCEBPB. Cells were loaded onto the upper compartments of Matrigel (30 μg/ml)-coated plates and incubated for 16 h. After fixing and staining with 0.6% hematoxylin and 0.5% eosin, the number of migrated or invasive cells was counted. All of the images are representative of at least five independent experiments

Honokiol effectively downregulated the protein expression of HDAC3, EMT markers (Snail, Slug, and Twist), CEBP/β, and phosphorylated NF-κB-p65, but not HDAC1/2, phosphorylated NF-κB-p52 or p50, and markedly upregulated ER stress-related markers (phosphorylated elf2α and GADD153) in gastric cancer cells (Fig. 7A). Knockdown HDAC3 by gene silencing could also suppress EMT markers protein expression and increase Occludin (an epithelial marker), but not E-cadherin (another epithelial marker), protein expression (Fig. 7B). Hence, we further examined whether E-cadherin distribution exchanged using immuno-histochemistry analysis. Exposed to Honokiol, HDAC3 inhibitor RGFP966, and gene silencing of HDAC3 markedly increased the expression of phosphorylated E-cadherin, but decreased the expression of β-catenin in SCM1 cells (Fig. 7C). We next examined whether knockdown of NF-κB-p65 and C/EBPβ participated in the EMT process in gastric cancer cells. Results revealed that TGFβ induced the changes of morphologic characteristics of epithelial cells, from the typical cobblestone type to the slender fibroblast-like, or exhibited a single spindle-shaped cell phenotype, which could be effectively reversed by the treatments with Honokiol, melatonin, and gene silencing of NF-κB-p65 or C/EBPβ (Fig. 7D). Quantification analysis was shown in Supplementary Fig. 4. We further verified the effect of Honokiol on cell invasion. Results showed that Honokiol, melatonin, and gene silencing of NF-κB-p65 or C/EBPβ markedly suppressed TGFβ-induced cell invasion (Fig. 7E). These results suggest that Honokiol is capable of inhibiting cell invasion via inhibition of NF-κB-p65 and C/EBPβ signals.

### Honokiol impeded cell migration and invasion via HDAC3 suppression in gastric cancer cells

**Fig. 8:**
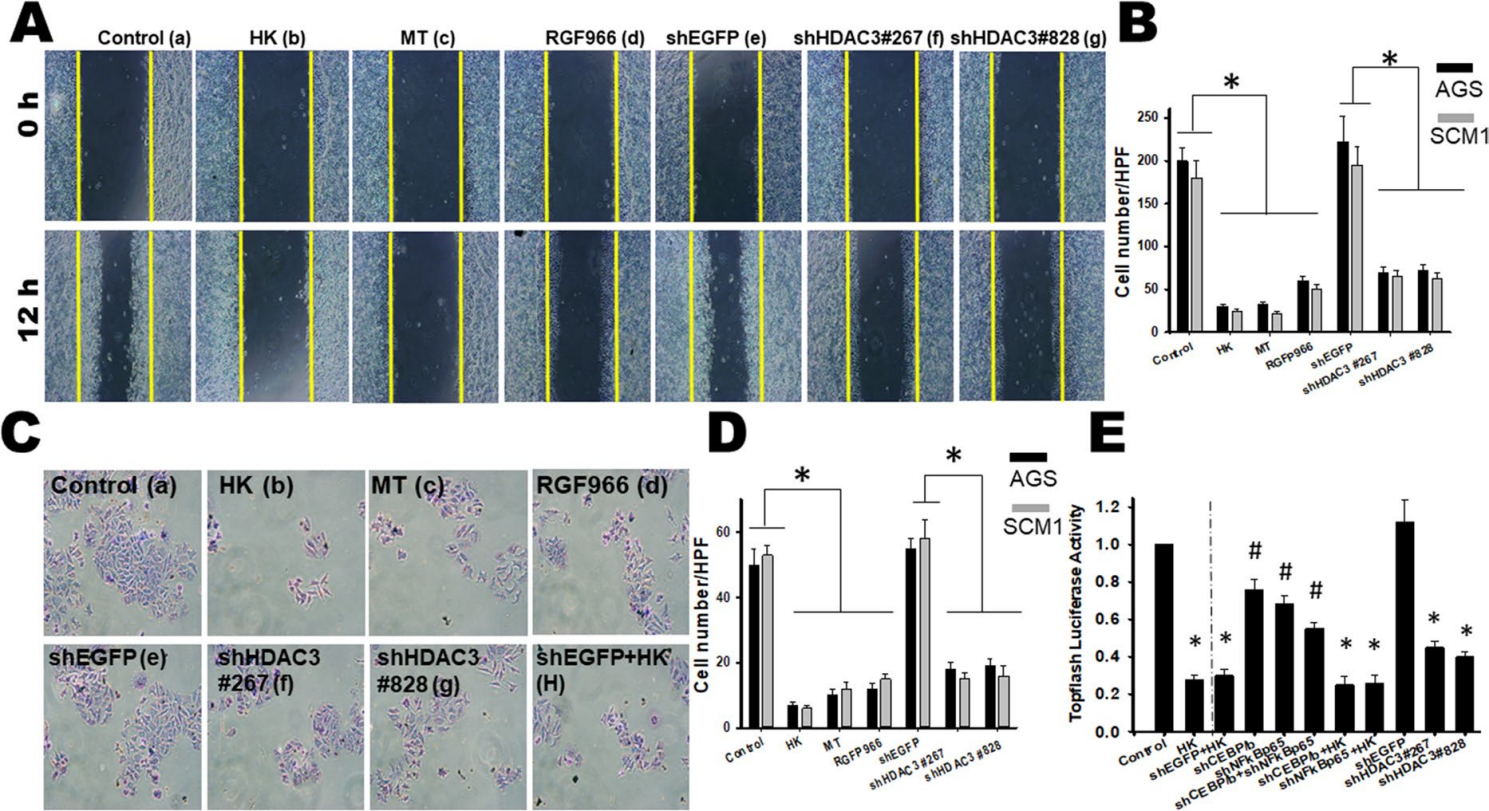
Suppression of HDAC3 by Honokiol-inhibited cell migration and invasion in gastric cancer cells. **A** Wound healing cell migration assays performed on monolayer cells revealed that Honokiol (HK), melatonin (MT), shHDAC3, and pharmacologic inhibitors RGFP966 mediated the inhibition of cellular migration in SCM1 cells. The graph shows the results of wound closure 18 h after wounding, as derived from six independent experiments. Melatonin was the positive control. **B** Quantifications of numbers of migration in different treatments (counts/field) were calculated. All data are presented as mean ± SD (*n* = 6). *p < 0.05 versus control cells. **C** For the cancer cell invasion assay, the numbers of migrated or invasive cells were counted after treatment. **D** Quantification of numbers of invasions in different treatments (counts/field) was made. All data are presented as mean ± SD (*n* = 6). *p < 0.05 compared with control. Cancer cells were co-transfected with TOPFlash or FOPFlash luciferase reporter genes along with Renilla luciferase. After 6 h, cells were left untreated (no treatment) or treated with Honokiol (HK) or as indicated silencing gene. After an additional 36 h, cells were harvested and luciferase levels were determined; firefly luciferase was normalized to Renilla. TOPFlash activity was highly induced in all six populations of cells and this was inhibited by Honokiol. As the negative control, FOPFlash showed minimal response to Honokiol. TOPFlash inhibition by Honokiol was more robust in the SCM1 than in the AGS cell line. The experiment was done five with similar results. #*p* < 0.05 and *p < 0.001 versus control cells. **E** Suppression of HDAC3 by Honokiol-inhibited cell migration, invasion and Wnt/ß-catenin activity in gastric cancer cells

To investigate if HDAC3 could confer enhanced migratory and invasive capacities to gastric cancer cells, the migration and invasion were assessed using two distinct shRNAs targeting HDAC3 (shHDAC3 #267; #828) and the pharmacologic HDAC3 inhibitor RGFP966. As controls, cells were transduced encoding a non-targeting shRNA (shEGFP). The efficiency of the shHDAC3-mediated silencing was confirmed by RT-qPCR, immuno-blotting, and HDAC3 activity (Supplementary Fig. 5). Honokiol, melatonin, RGF966, and shRNAs targeting HDAC3 significantly impaired cell migration (Fig. 8A and B) and invasion (Fig. 8C and D). Efficacy was confirmed by monitoring the number of viable cells. In both assays, HDAC3 silencing reduced the capacity of gastric cancer cells to migrate by more than 60%. Transwell invasion assays further revealed significant impairment of gastric cancer cells to invade through the Matrigel-coated filters when HDAC3 was silenced.

To explore the involvement of Wnt/β-catenin in ER stress-reduced peritoneal cavity, Wnt/β-catenin signaling was examined in gastric cancer cells. Results revealed that Honokiol significantly decreased the nuclear localization of β-catenin (data not shown). Honokiol also decreased the TopFlash activity for Wnt/β-catenin signaling (Fig. 8E). The gene knockdown of NFκB-p65, C/EBPβ, or HDAC3 combined with/without Honokiol treatment could also significantly decrease the TopFlash activity (Fig. 8E). Interestingly, shNFκB-p65, shC/EBPβ, or shHDAC3 prevented the down-regulation of TopFlash activity induced by the knockdown per se, suggesting that Wnt/β-catenin may be a target of NFκB-p65, C/EBPβ, or HDAC3 in vitro. These results also indicated that Honokiol significantly suppressed the mesenchymal characteristics, increased the expression of epithelial signature markers, and suppressed the Wnt expression through NFκB-p65-, C/EBPβ-, or HDAC3-dependent regulation, which affected cancer cell migration and invasion in vitro.

### Honokiol and gene silencing HDAC3 restrained gastric tumor growth and peritoneal dissemination in a mouse model

**Fig. 9:**
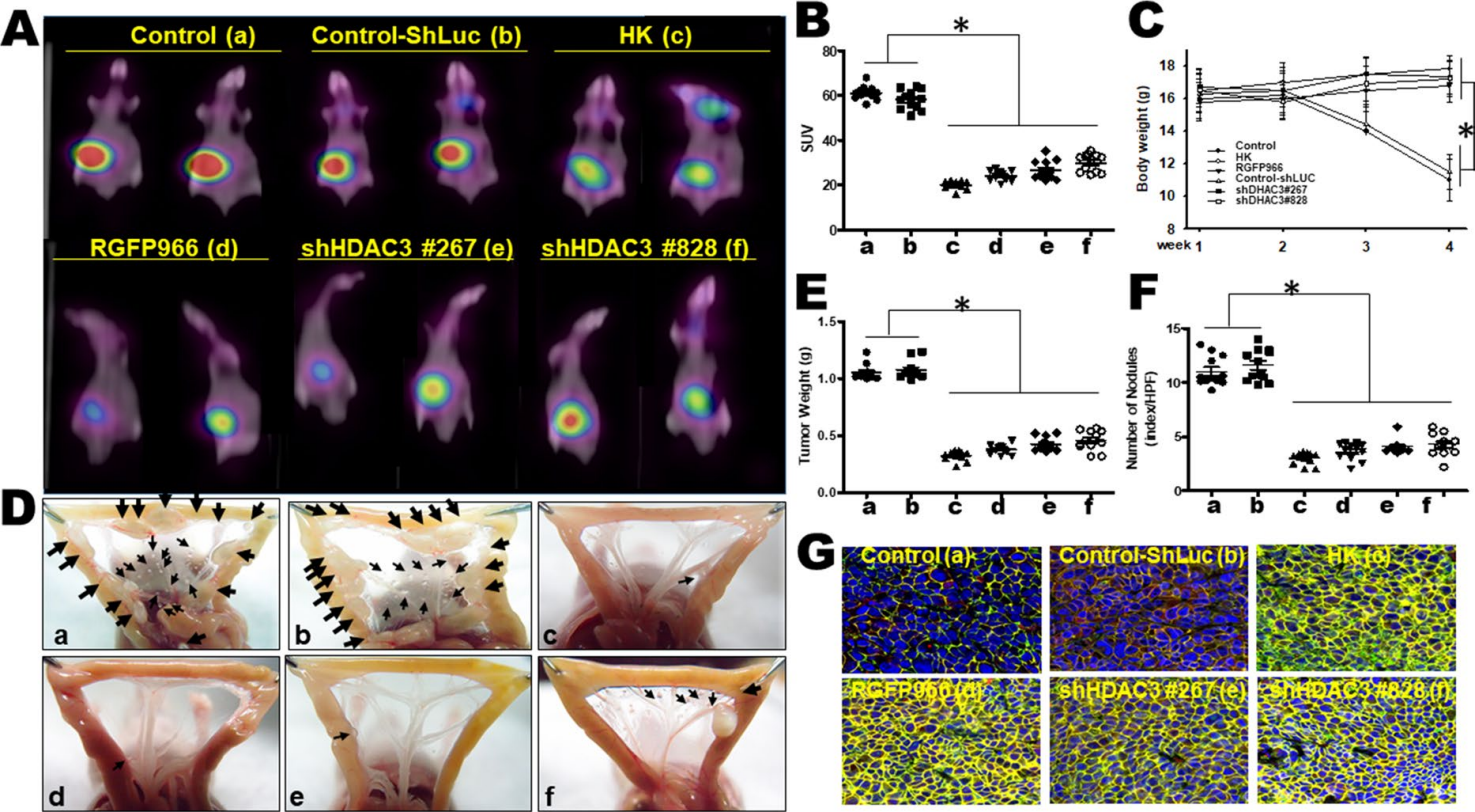
Treatments with Honokiol, HDAC3 gene silencing, or RGFP966 inhibited the metastasis of gastric tumors in a xenograft tumor mouse model. Mice received intra-peritoneal implants of MKN45 cells and were treated with Honokiol (5 mg/kg, twice/weekly, intra-peritoneal injection), RGFP966 (5 mg/kg, twice/weekly, intra-peritoneal injection) or knockdown HDAC3 expression (shHDAC3 #267; #828). **A** Randomized grouping of Honokiol-treated mice. Tumor burden was measured twice per week using PET/CT imaging. A representative image shows the peritoneal dissemination of animals. The results are representative of at least 8–12 independent experiments. **B** Quantifications of representative images showing peritoneal dissemination of therapy-treated animals and controls. All data are presented as mean ± SD. *n* = 8–12). *p < 0.05 versus control mice. **C** The body weight of all groups of mice was presented as a bar graph. All data are presented as mean ± SD (*n* = 8–12). *p < 0.05 compared to controls. **D** Photomacrographs of metastatic peritoneal nodules vision of animals inoculated with MKN45 gastric cancer cells with or without scramble, knockdown gene treatment (arrows shown). The maximum intensity image of nude mice (tumor control and scramble) showed many metastatic nodules in the mesentery of the control mice inoculated with MKN45 cells. In contrast, peritoneal metastatic nodules were observed sporadically in Honokiol treated or gene silencing shHDAC3 or RGFP966-treated or mice. **E** The weights of metastasis mesenteric tumors are presented as a bar graph. **F** Quantifications of nodule number. All data are presented as mean ± SD (*n* = 8–12). *p < 0.05 versus control mice. **G** Animal tissues staining for Honokiol, HDAC3 inhibitor RGFP966, and gene silencing of HDAC3 expression (shHDAC3 #267; #828) probed with monoclonal antibodies against specific p-E-cadherin (green), β-Catenin (red), and Hoechst (blue). Confocal images for p-E-cadherin, β-Catenin, and Hoechst were examined and quantified as described in the “Materials and methods” section. The images are representative of at least six independent experiments. The average intensity per cell is presented as mean ± SD

Honokiol administration (5 mg/kg/twice/weekly) inhibited tumor growth, metastatic peritoneal extension, and macro-metastasis by 80–85% compared to the control group detected by PET/CT imaging and quantification (Fig. 9A and B) and macroscopic examination of the tumor burden (Fig. 9D) along with major organ metastasis (Supplementary Fig. 6). Quantifications of estimated radioactivity (Bq/ml) and specific uptake values (SUV), which were as indices to determine if a hotspot was significant, were calculated. Gene silencing HDAC3 treatment repressed intestinal mesenteric nodules and had diverse organs effects similar to those of the pharmacologic inhibitor RGFP966 (Supplementary Fig. 6). Quantification of parietal peritoneum metastasis of gastric tumor cells by Honokiol and gene silencing HDAC3 revealed that Honokiol had no toxic effects and had modest gains in body weight. The quantifications of body weight, tumor weight, and a number of nodules were shown in Fig. 9C–F, respectively. Histological examination for animal tumor tissues revealed high expression levels of E-cadherin phosphorylation in the groups of Honokiol and gene silencing HDAC3 (Fig. 9G). These results indicated that Honokiol therapy led to a substantial diminution in gastric tumor growth and metastatic dissemination in an experimental mouse model.

## Discussion

**Fig. 10:**
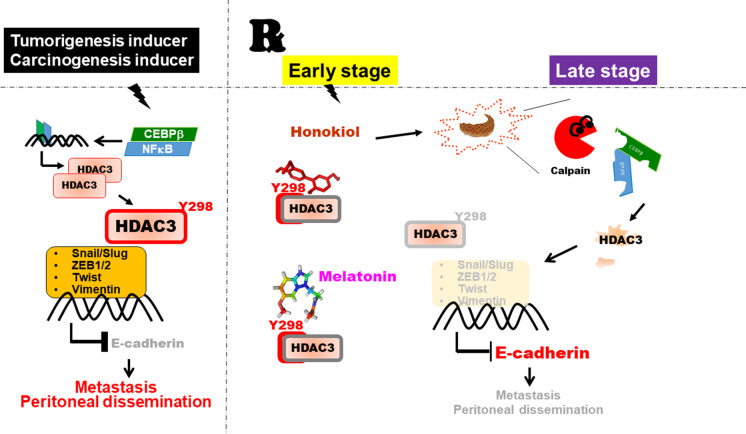
Conceptual model showing the working hypothesis on the interaction among Honokiol, HDAC3, and ER stress in the peritoneal dissemination of gastric cancer. In the schematic of the proposed mechanism for the role of Honokiol targeting HDAC3 by ER stress cascade and mitigating the peritoneal spread of gastric cancer. Honokiol-induced ER stress–activated calpain activity targeted HDAC3 and blocked Tyr298 phosphorylation, subsequently blocked cooperating with EMT transcription factors and cancer progression. The present study provides evidence to demonstrate that HDAC3 is a positive regulator of EMT and metastatic growth of gastric cancer cells. The findings here imply that overexpressed HDAC3 is a potential therapeutic target for honokiol to reverse EMT and prevent gastric cancer migration, invasion, and metastatic dissemination

The findings of the current study support the hypothesis that HDAC3 plays a crucial role in suppressing gastric cancer cell metastatic dissemination, which can be modulated by Honokiol-induced ER stress. Honokiol also promotes calpain activation by targeting NF-κB-p65 and C/EBPβ, thereby inhibiting the effects of HDAC3. Moreover, HDAC3 is significantly over-expressed in both gastric tissues and gastric cancer cell lines. Histopathologic investigation of human gastric cancer tissues reveals a significant correlation between HDAC3 expression and clinical stages, expressed in the TNM (Tumor-Node-Metastasis) classification. In the analysis of verified clinical samples of the omentum, distal, and lymph node metastases, high HDAC3 expression with markedly dense staining is a poor prognostic indicator in patients with gastric cancer. In animal experiments, HDAC3 overexpression in tumors can be significantly inhibited by Honokiol treatment. Furthermore, the downregulation of HDAC3 through gene silencing markedly blocks the metastatic capability of gastric cancer cells in nude mice, especially towards the abdominal cavity, liver, and lungs. These findings demonstrate that HDAC3 detection may provide clear and realistic explanations regarding useful information, while histopathologic studies may provide prognostic stratification and help in choosing appropriate treatment strategies (Fig. 10).

Class I histone deacetylase HDAC3 is involved in “epigenetic” gene regulation through the control of the acetylation state of lysine side chains in histone tails. HDAC3 is also a component of the NCoR-SMRT co-repressor complex that is different from co-repressor complexes, which typically contain HDAC1 and 2 (Watson et al. 2016). A previous report showed that the HDAC3 protein had common features with HDAC1 and HDAC2, namely deacetylation of histone substrates and transcriptional repression in early functional analysis (Karagianni and Wong 2007; Yang et al. 1997). In contrast, HDAC3 includes an intriguingly variable C terminus, with no apparent similarity to other HDACs, indicating that HDAC3 may have some unique properties and may not be completely redundant with other HDACs. Moreover, advanced functional domain analysis reveals that HDAC3 contains both nuclear export and import signals, which account for its distinct localization pattern (Yang et al. 2002). The subcellular distribution of HDAC3 supports its nuclear function and uncovers a whole new world of potential cytoplasmic substrates and regulators. Initial implications of HDAC3 in tumorigenesis and carcinogenesis have been based on correlative studies reporting aberrant expression and/or localization of HDAC3 in various tumors (Bartling et al. 2005). Elevated and deregulated HDAC3 expression is also found in human astrocytic glial tumors (Liby et al. 2006). Gene silencing of HDAC3 in human colon cancer results in cell growth inhibition, differentiation, and increased apoptosis (Wilson et al. 2006). From a mechanistic perspective, both epithelial and mesenchymal cells respond to hypoxia with a HIF-dependent induction of HDAC3. In the epithelia, increased HDAC3 deacetylates H3K4ac to restrain the chromatin structure and expression of epithelial genes. At the same time, both WDR5 and HDAC3 levels are increased in mesenchymal cells. A previous study has shown that an interplay between HDAC3 and WDR5 collectively regulates low levels of oxygen-induced EMT (Mani and Barton 2011). Furthermore, the tumor antigen MAGE-A can recruit HDAC3 to impair the trans-activation function of the tumor suppressor p53 that confers resistance to chemotherapeutic agents (Monte et al. 2006). Based on a study with human maxillary carcinoma cells under acidic conditions, HDAC3 inhibition combined with hyperthermia may provide an efficient therapeutic approach for cancer (Narita et al. 2005).

Previous studies have shown a contributing role of HDAC3 in the compendium of clinical cancers. However, the role of HDAC3 in cancers is controversial. In a study, HDAC3 depletion substantially increased the migration of MDA-MB-231 metastatic breast cancer cells (Kim et al. 2010). Loss of HDAC3 could lead to hepatocellular carcinoma (Bhaskara et al. 2010), while another study found that the increased HDAC3 levels are associated with improved patient survival (Junqueira-Neto et al. 2015). In line with the results reported by Wu et al. (2011), knockdown of endogenous HDAC3 caused a complete and significant loss of EMT and inhibited the migration and invasion activity in FADU or MCF-7 clones. A critical role of HDAC3 in facilitating the control of EMT by different EMT regulators has been suggested (Mani and Barton 2011; Wu et al. 2011). Similarly, the current study also revealed that HDAC3 could interact with different EMT regulators to mediate E-cadherin repression in gastric cancer cells. Indeed, the immuno-histochemical evidence is consistent with the aforementioned findings and demonstrates that the increased HDAC3 protein levels lead to worse clinical outcomes in patients with gastric cancer.

EMT contributes to early metastasis, tumor progression, and resistance to treatments, indicating the development of cancer aggressiveness, growth, and spread in the early stages of the disease, although the role of EMT in metastasis is a longstanding source of debate (Cheung et al. 2013; Fischer et al. 2015; Zheng et al. 2015). Understanding the regulatory factors for EMT may help in the development of anti-metastasis therapeutics, anti-chemoresistance, as well as diagnostic or prognostic markers for various tumors. However, the effects of HDACs on EMT are also controversial. Numerous studies have demonstrated that HDACs inhibit EMT but this is not always the case. It is noteworthy that HDAC inhibitors might reinforce EMT progression (Debeb et al. 2012; Ji et al. 2015; Kong et al. 2012; Sakamoto et al. 2016; Wawruszak et al. 2019a, b; Wu et al. 2016a). Although the role of different HDACs in mediating EMT is well documented, whether or not a transcription-regulated HDAC3 is required to coordinate different EMT regulators remains unknown. As previously reported by Qi et al. (2008), HDAC3 is related to Snail signaling and HDAC inhibitors impair Snail-mediated transcriptional repression in cells during early Drosophila development (Qi et al. 2008). In addition, Zeng and colleagues (Zeng et al. 2013) have demonstrated that HDAC3 mRNA can undergo unconventional splicing to modulate HDAC3 and induce EMT via transforming growth factor β2 (TGFβ2) (Zeng et al. 2013). Zhang et al. (2019) have also reported that HDAC3 down-regulation by ginsenoside Rg3 inhibits the EMT of cutaneous squamous cell carcinoma via c-Jun acetylation (Zhang et al. 2019). Recently, I-7ab has been found to inhibit the growth of triple-negative breast cancer (TNBC) cells via targeting HDAC3 and promoting p53 acetylation (Yang et al. 2018). The miRNA regulates hypoxia-induced EMT and metastasis by repressing HDAC3 and SENP1 expression and by presenting a regulatory network, which involves many key players in hypoxia-induced EMT (Chen et al. 2016). The RORα/HDAC3-mediated attenuation of NF-κB signaling controls the balance of inflammatory responses, suggesting that therapeutic strategies targeting this epigenetic regulation may be beneficial in the treatment of chronic inflammatory diseases, including inflammatory bowel disease (IBD) (Oh et al. 2019). Wang et al. (2016) showed that disrupting the CBX4-HDAC3 interaction abolished the Runx2 inhibition, as well as the inhibition of cell migration and invasion (Wang et al. 2016). Clinically, compared to normal adjacent tissues, esophageal tumor samples showed an up-regulation of SOX4, EZH2, and HDAC3 in which the EZH2 expression was significantly increased in metastatic ESCC tissues (Koumangoye et al. 2015). SOX4 promotes esophageal tumor cell proliferation and invasion by silencing miR-31 via the activation and stabilization of a co-repressor complex with EZH2 and HDAC3 (Koumangoye et al. 2015). In the present study, the findings suggest a crucial role for HDAC3 in suppressing E-cadherin transcription and in up-regulating mesenchymal markers during EMT in gastric cancer.

Honokiol is a small-molecule polyphenol, which has been shown to be a potential anticancer agent in multiple facets of signal transduction and molecular action. Wang et al. have demonstrated that Honokiol inhibits breast cancer cell metastasis by blocking EMT through the modulation of Snail/Slug protein translation (Wang et al. 2019). Avtanski et al. have shown that Honokiol attenuates EMT by targeting STAT3/Zeb1/E-cadherin axis in breast cancer cells (Avtanski et al. 2014). Singh et al. have also found that treatment of non-small cell lung cancer cells with Honokiol suppresses the levels of class I HDAC proteins and HDAC activity while enhances the HAT activity in vitro and in vivo (Singh et al. 2013). Our findings are partly consistent with these previous studies. In the present study, except for the detection of HDAC3 activity, the gold standard radioligand binding assay and differential scanning calorimetry (DSC) were further used to identify the affinity of ligand binding to a target receptor, due to their robustness and sensitivity (Fig. 2 and Supplementary Fig. 1A). Moreover, the levels of both protein and mRNA expression for HDAC3 were also determined (Supplementary Fig. 1D-6E). Western blot analysis revealed that treatment of cells with Honokiol resulted in a time-dependent increase in the levels of the acetylated histone H3 and histone H4 compared to the vehicle-treated control cells. (Supplementary Fig. 1F). Screening by molecular docking, a key tool in structural molecular biology and computer-assisted drug design, assessed the novel functional site at Tyr298-HDAC3 for Honokiol (Fig. 3B–E). Determined specific phospho-HDAC3 (Tyr298) by western blot convinced the evidence simultaneously (Fig. 3F). We also ruled out the involvement of HDAC1/2 in the Honokiol-induced effects on gastric cancer cells (Figs. 6A and 7A and Supplementary Fig. 1). This study provides novel insights into the molecular mechanisms of Honokiol that can regulate the epithelial-mesenchymal plasticity and metastatic dissemination in gastric cancer.

However, the present study still has some limitations. Does Honokiol affect the HDAC3 function? Some HDACs are known to possess substrate specificity, but HDACs may also act to deacetylate nonhistone proteins. The effects of Honokiol on HDAC3 function may need to be clarified. It may also need to clarify that the effects of Honokiol on proliferation and apoptosis in gastric cancer cells, which may also explain the reduction in migration and metastasis. Moreover, although the effects of Honokiol and HDAC3 on both ER stress response and EMT have been observed, it remains unclear if honokiol is only working through HDAC3 to mediate these effects. The definitive rescue experiments linking HDAC3 with Honokiol may need to use to clarify the effects of HDAC3 with those of honokiol remains.

## Summary and conclusion

The present study demonstrates that HDAC3 can regulate E-cadherin expression and EMT through the transcription factors C/EBPβ and NFκB. Our data also reveal that HDAC3 knockdown downregulates the expression of Wnt/β-catenin signaling. To determine if transcription factors C/EBPβ and NFκB have any causal role in Wnt/β-catenin signaling, silencing of C/EBPβ and NFκB individually has been done. The results showed that the Wnt/β-catenin activity by TopFlash luciferase assay was decreased in gastric cancer cells with gene silencing of C/EBPβ and NFκB-p65, indicating that Wnt/β-catenin may be a target for NFκB-p65 or C/EBPβ. The pharmacologic inhibition or gene silencing of HDAC3 could also suppress the migration and metastasis in gastric cancer cells in vitro and in vivo. The experimental animal model also proves a HDAC3 knockdown-mediated epithelial morphology accompanied by increased E-cadherin, suggesting a pathologic role of HDAC3 regulation in EMT expression. Nevertheless, how HDAC3 can regulate the expression of these EMT-inducing transcription factors and Wnt/β-catenin signaling in gastric cancer cells warrants further investigation. Overall, this study demonstrates that HDAC3 may be a positive regulator of EMT and tumor metastasis in gastric cancer through transcriptional repression of E-cadherin via C/EBPβ and NFκB-p65 gene regulation, which can be significantly inhibited by treatment with Honokiol or HDAC3 inhibitors. Thus, HDAC3 may be a potential therapeutic target for reversing EMT and tumor metastasis in gastric cancer progression.

### Supplementary Information

Below is the link to the electronic supplementary material.

Supplementary Fig. 1 (PNG 19.3 MB)

High resolution image (TIF 177 KB)

Supplementary Fig. 2 (PNG 19.3 MB)

High resolution image (TIF 138 KB)

Supplementary Fig. 3 (PNG 25.7 MB)

High resolution image (TIF 150 KB)

Supplementary Fig. 4 (PNG 25.7 MB)

High resolution image (TIF 392 KB)

Supplementary Fig. 5 (PNG 25.7 MB)

High resolution image (TIF 135 KB)

Supplementary Fig. 6 (PNG 25.7 MB)

High resolution image (TIF 1200 KB)

Supplementary Fig. 7 (PNG 19.3 MB)

High resolution image (TIF 151 KB)

Supplementary Fig. 8 (PNG 19.3 MB)

High resolution image (TIF 89 KB)

## Data Availability

Data is available on request from the authors. The data that support the findings of this study are available from the corresponding author upon reasonable request. Some data may not be made available because of privacy or ethical restrictions.

## Abbreviations

GC: Gastric cancer
HDIs: Histone deacetylase inhibitors
HDAC3: Histone deacetylase 3
EMT: Epithelial-mesenchymal transition
ER: Endoplasmic reticulum
C/EBPβ: CCAAT/enhancer-binding protein B
NF-κB: Nuclear factor kappa-light-chain-enhancer of activated B cells
ERSE: ER stress response element
TEM: Transmission electron microscope
PET/CT: Positron emission tomography-computed tomography
shRNA: Short hairpin RNA
DSC: Differential scanning calorimetry
NBD: Nucleotide-binding domain
Tm: Melting temperature
