# Supplementary figures and images for: Targeting histone deacetylase-3 blocked epithelial-mesenchymal plasticity and metastatic dissemination in gastric cancer
Source: Cell Biol Toxicol. 2022 Jan 1;39(5):1873–96. doi: 10.1007/s10565-021-09673-2 (PMC10547655; doi:10.1007/s10565-021-09673-2)

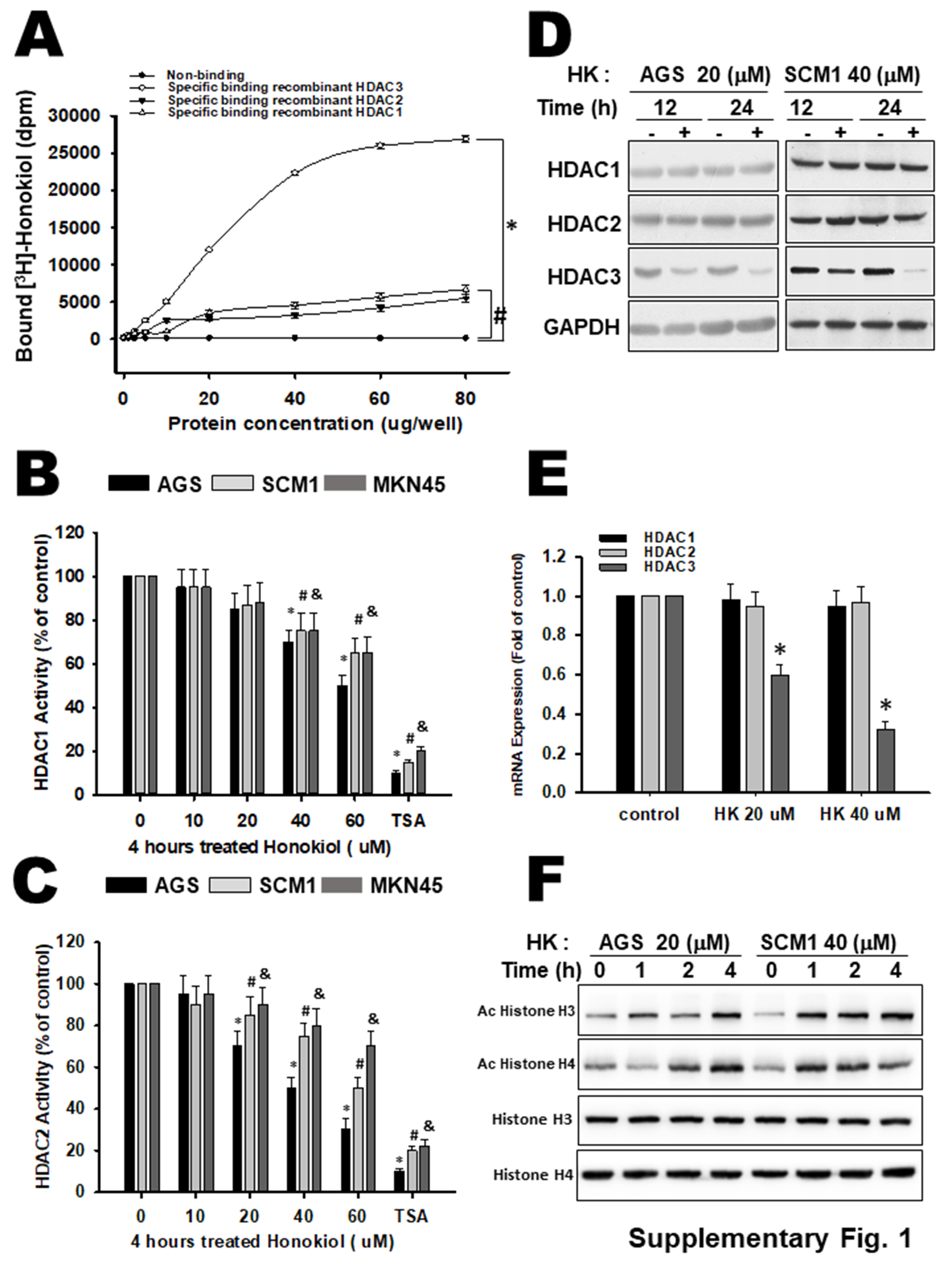

Supplement: Supplementary file 1 — Supplementary Fig. 1 (PNG 19.3 MB) [file 10565_2021_9673_Fig11_ESM.png]

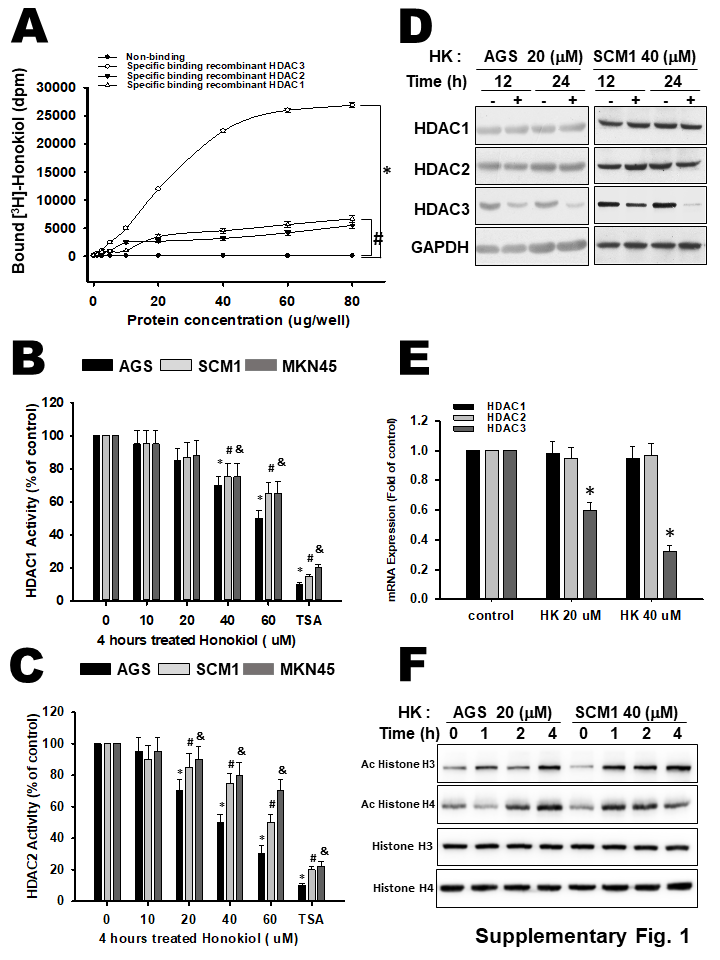

Supplement: Supplementary file 2 — High resolution image (TIF 177 KB) [file 10565_2021_9673_MOESM1_ESM.tif]

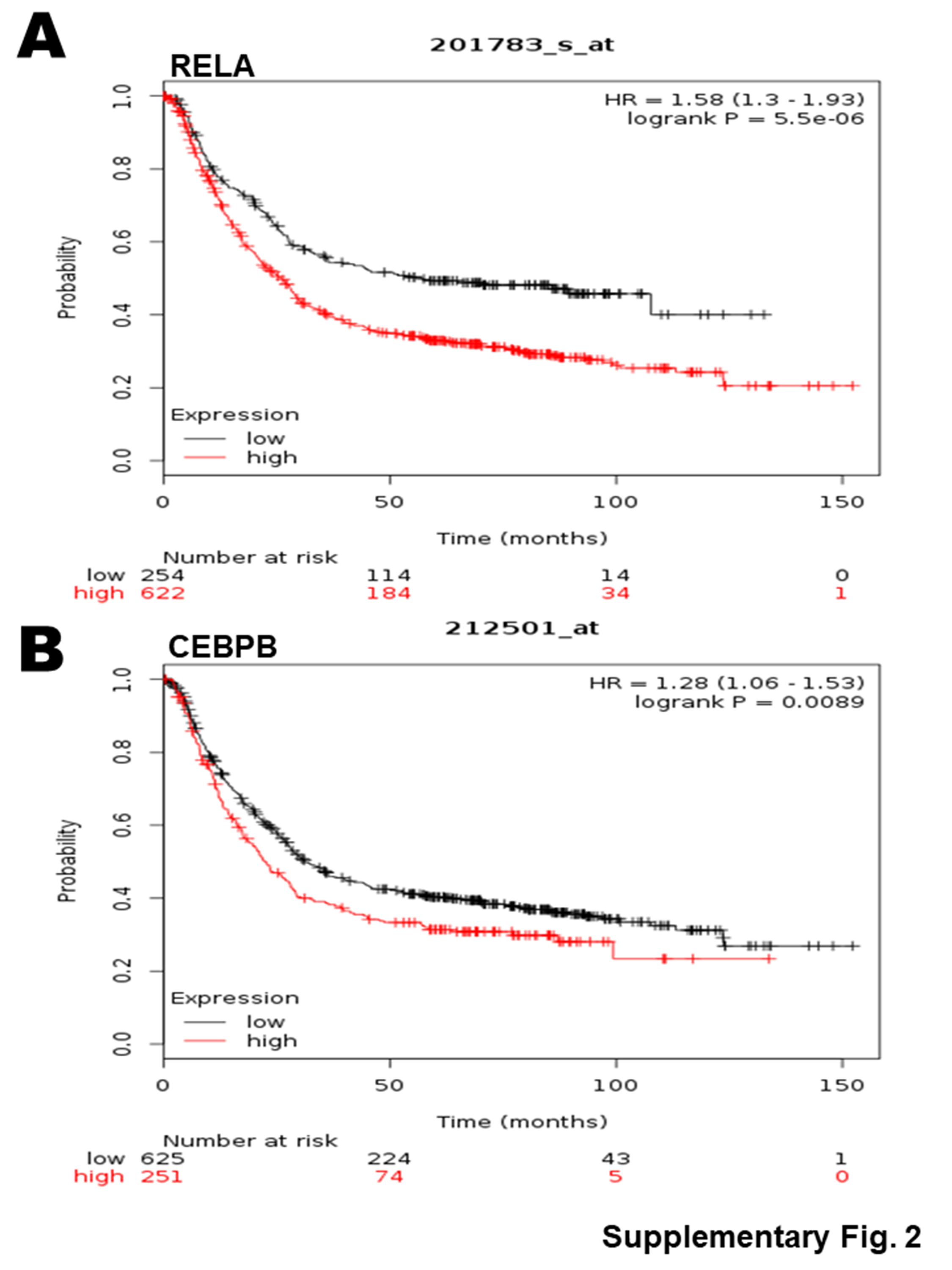

Supplement: Supplementary file 3 — Supplementary Fig. 2 (PNG 19.3 MB) [file 10565_2021_9673_Fig12_ESM.png]

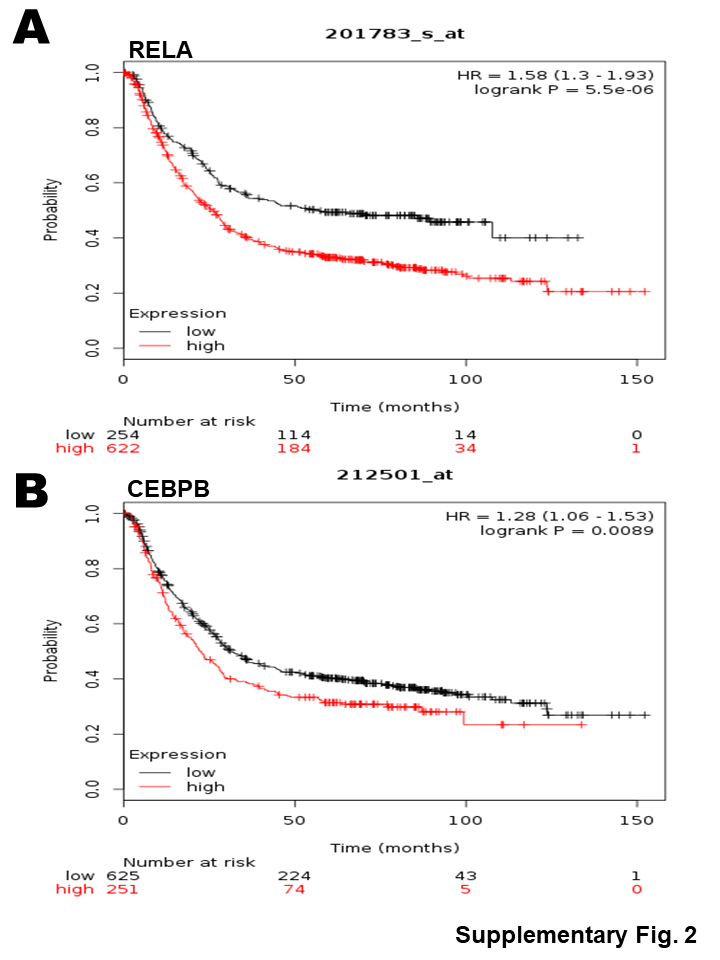

Supplement: Supplementary file 4 — High resolution image (TIF 138 KB) [file 10565_2021_9673_MOESM2_ESM.tif]

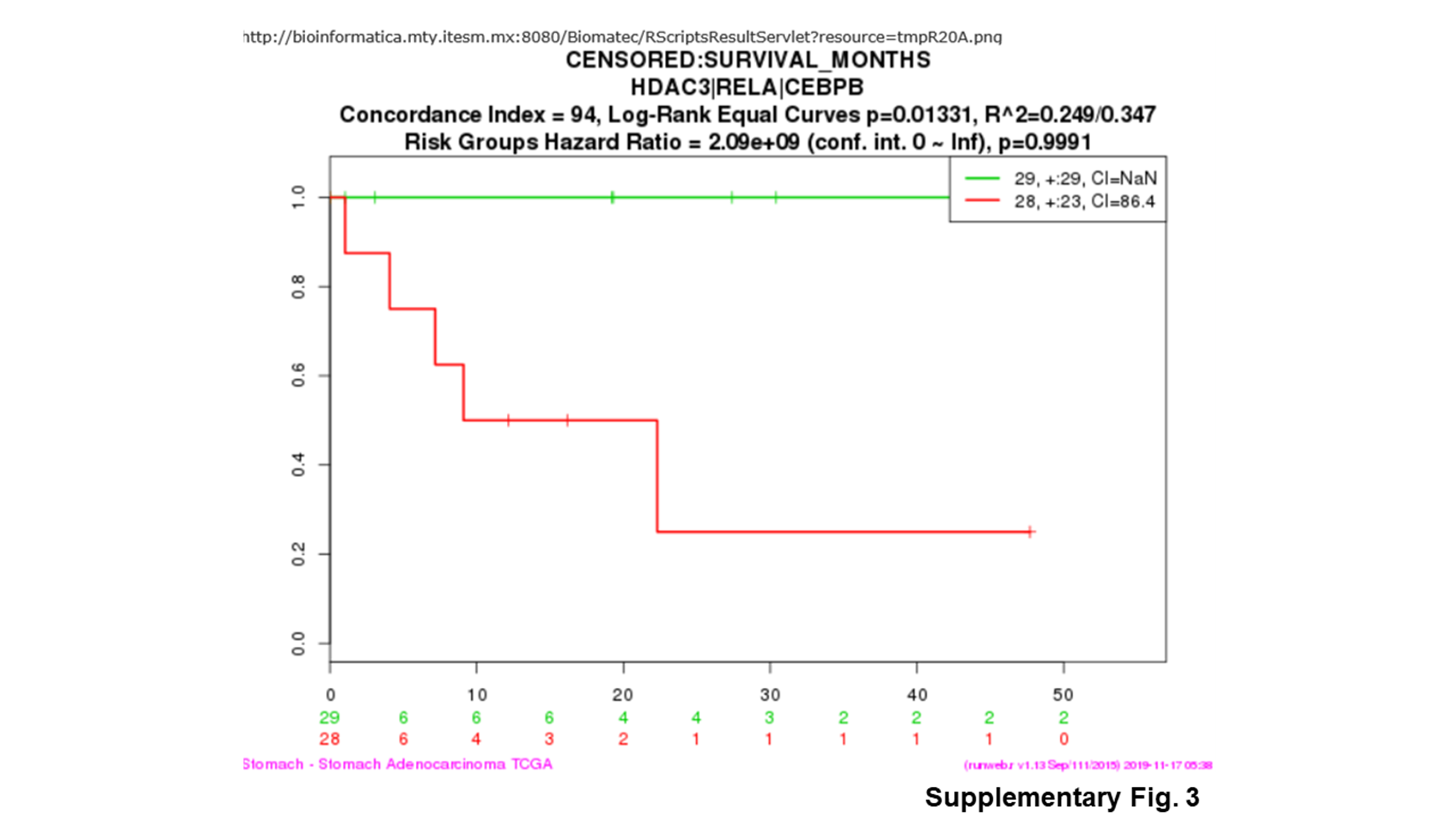

Supplement: Supplementary file 5 — Supplementary Fig. 3 (PNG 25.7 MB) [file 10565_2021_9673_Fig13_ESM.png]

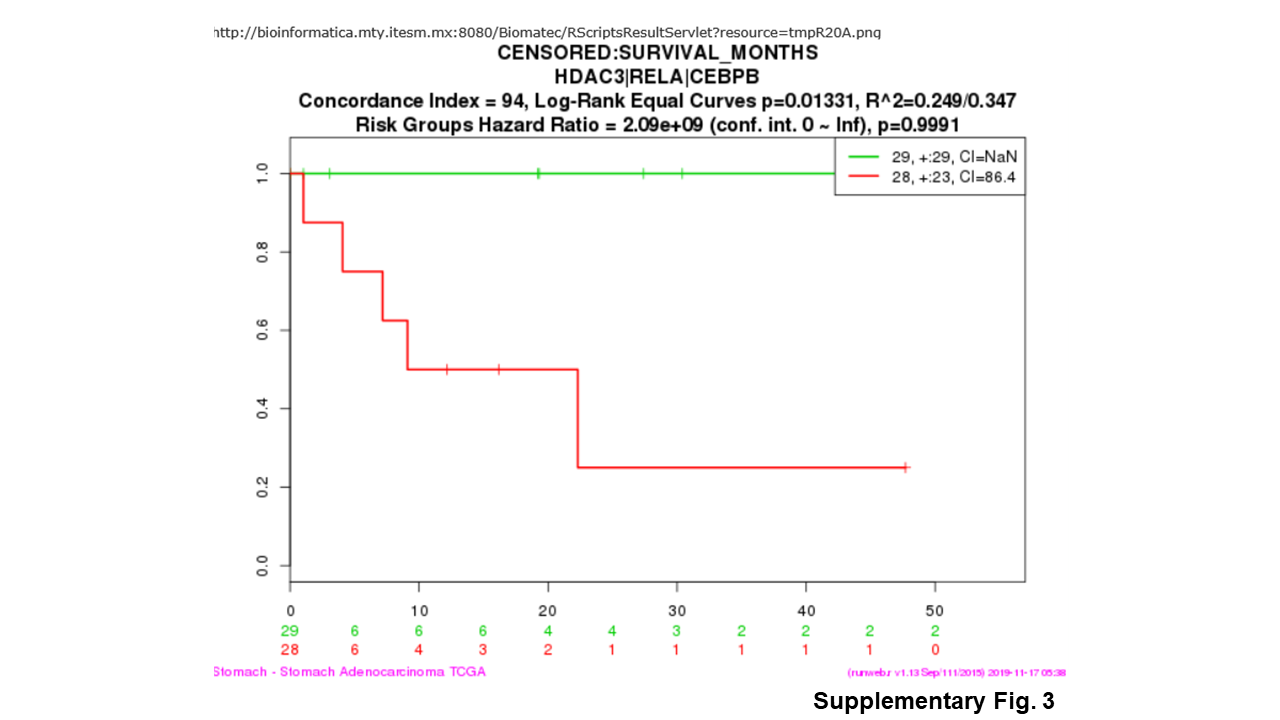

Supplement: Supplementary file 6 — High resolution image (TIF 150 KB) [file 10565_2021_9673_MOESM3_ESM.tif]

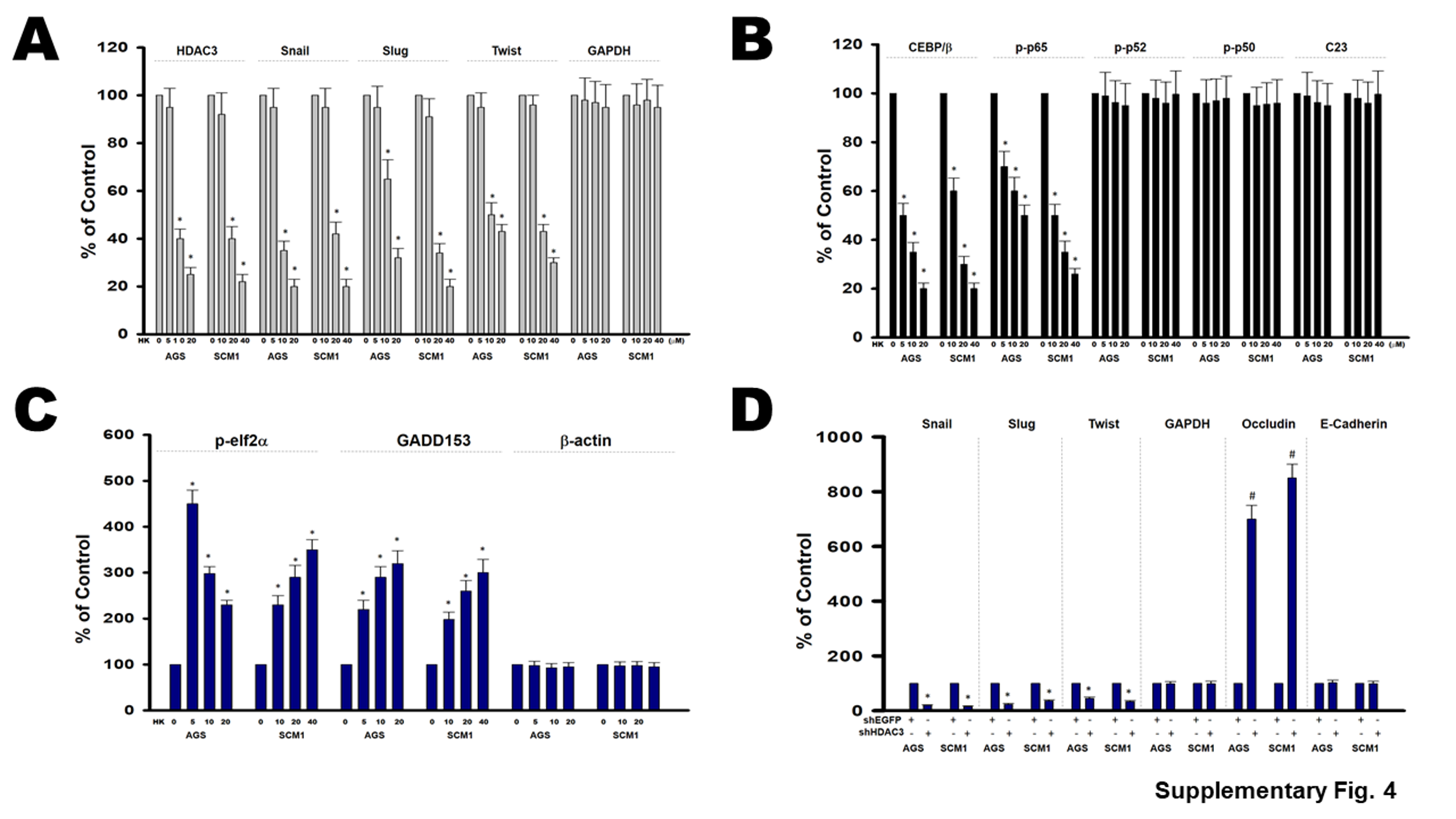

Supplement: Supplementary file 7 — Supplementary Fig. 4 (PNG 25.7 MB) [file 10565_2021_9673_Fig14_ESM.png]

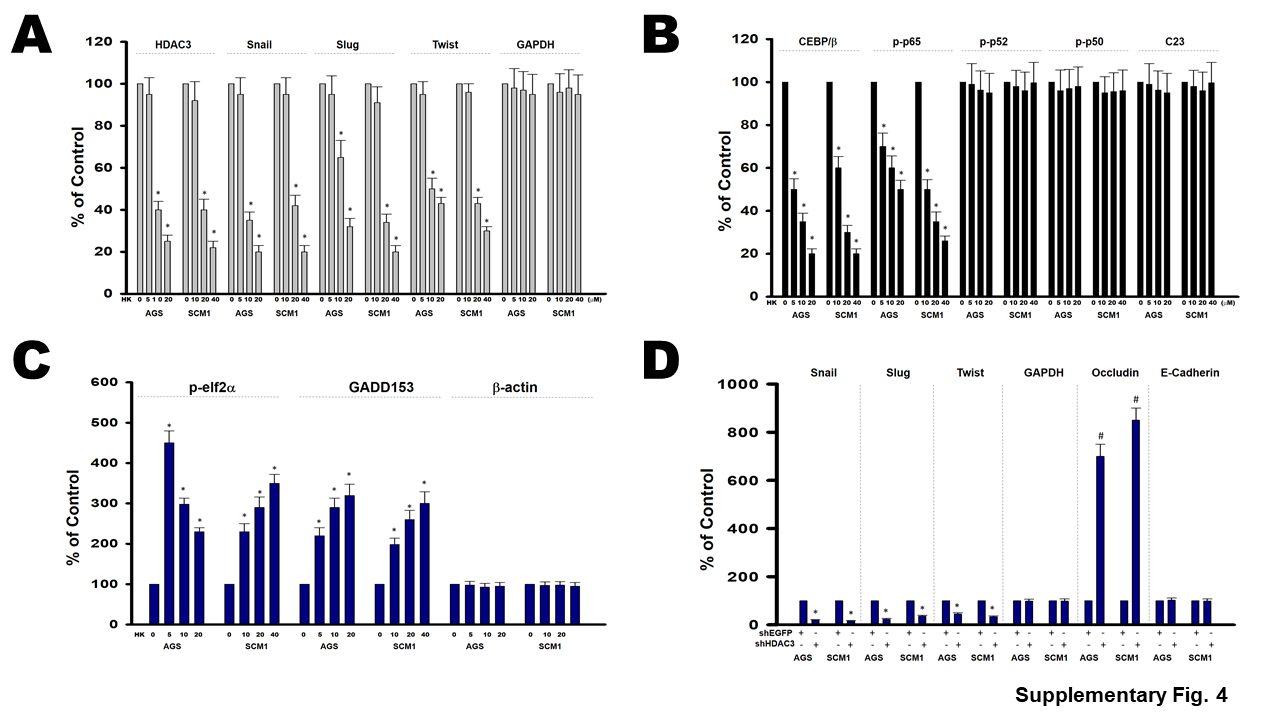

Supplement: Supplementary file 8 — High resolution image (TIF 392 KB) [file 10565_2021_9673_MOESM4_ESM.tif]

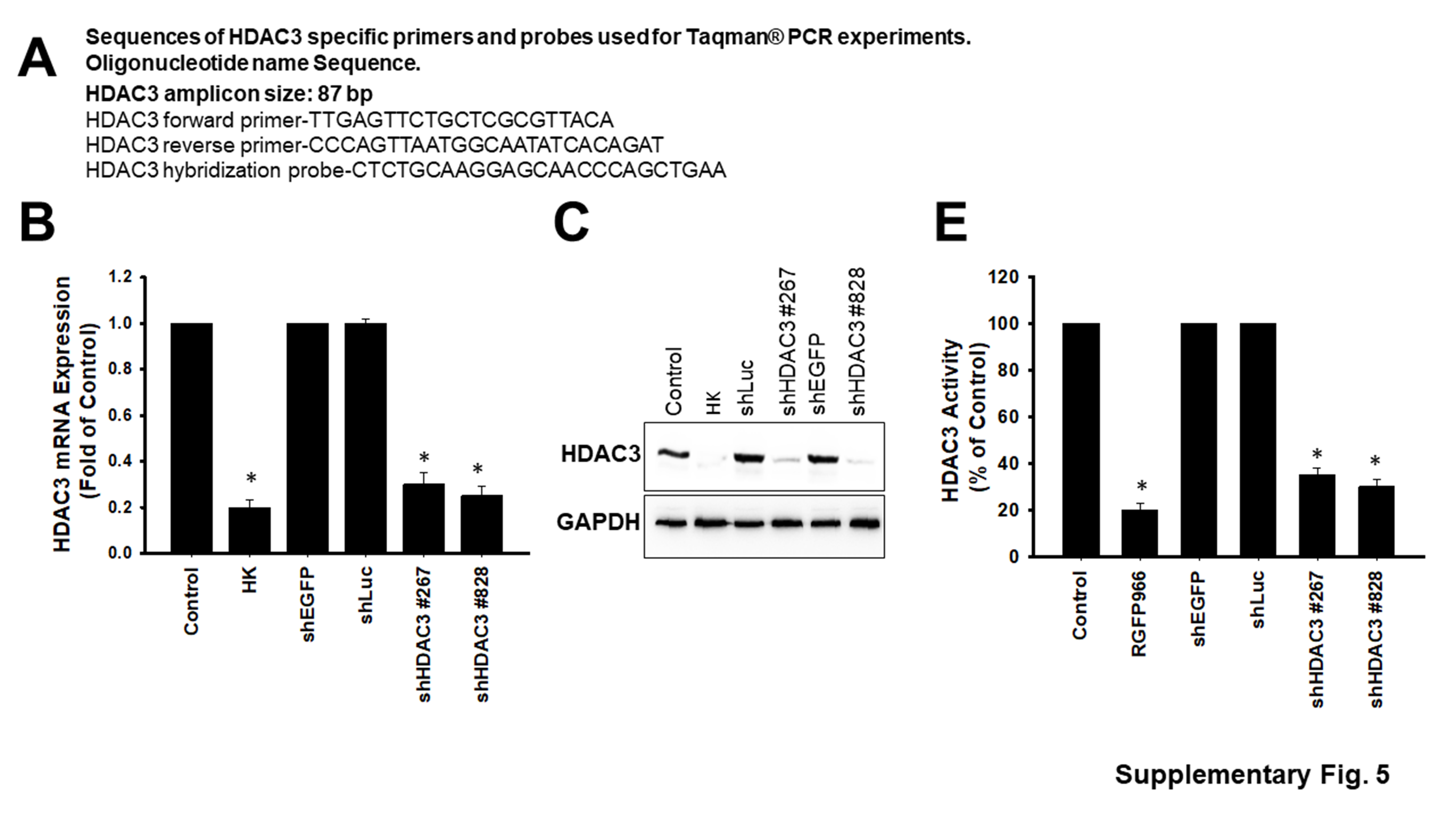

Supplement: Supplementary file 9 — Supplementary Fig. 5 (PNG 25.7 MB) [file 10565_2021_9673_Fig15_ESM.png]

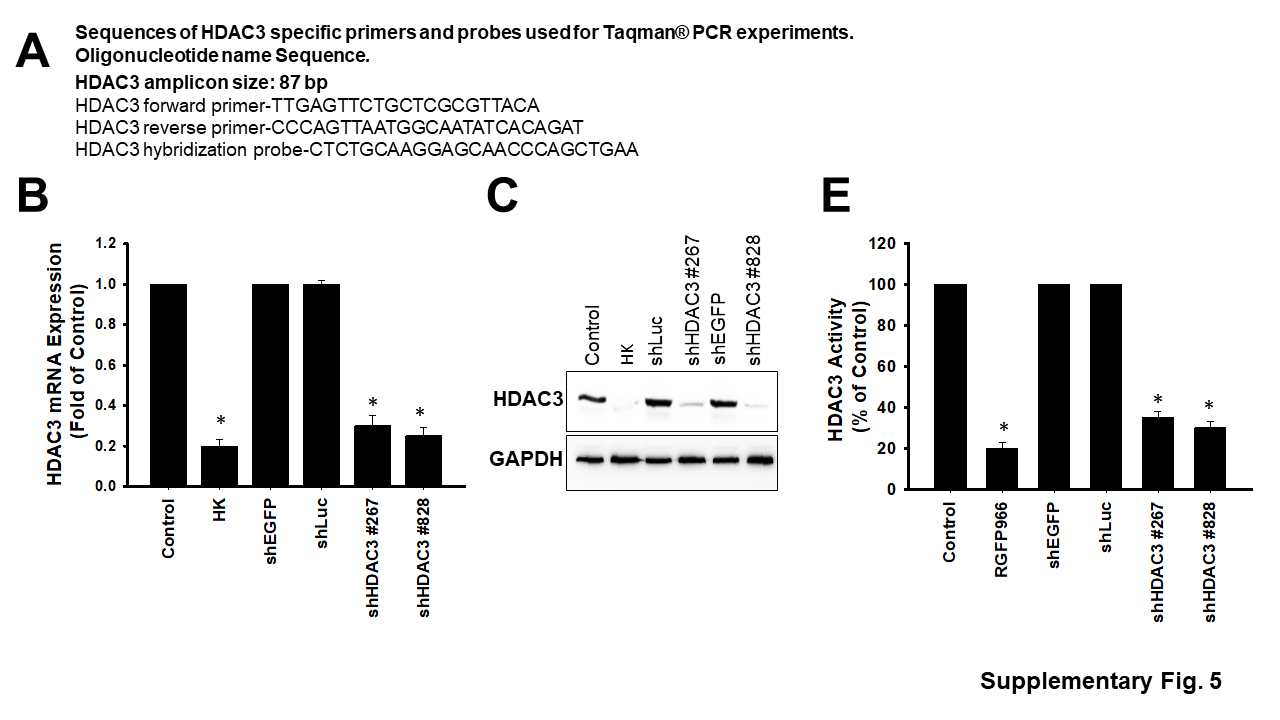

Supplement: Supplementary file 10 — High resolution image (TIF 135 KB) [file 10565_2021_9673_MOESM5_ESM.tif]

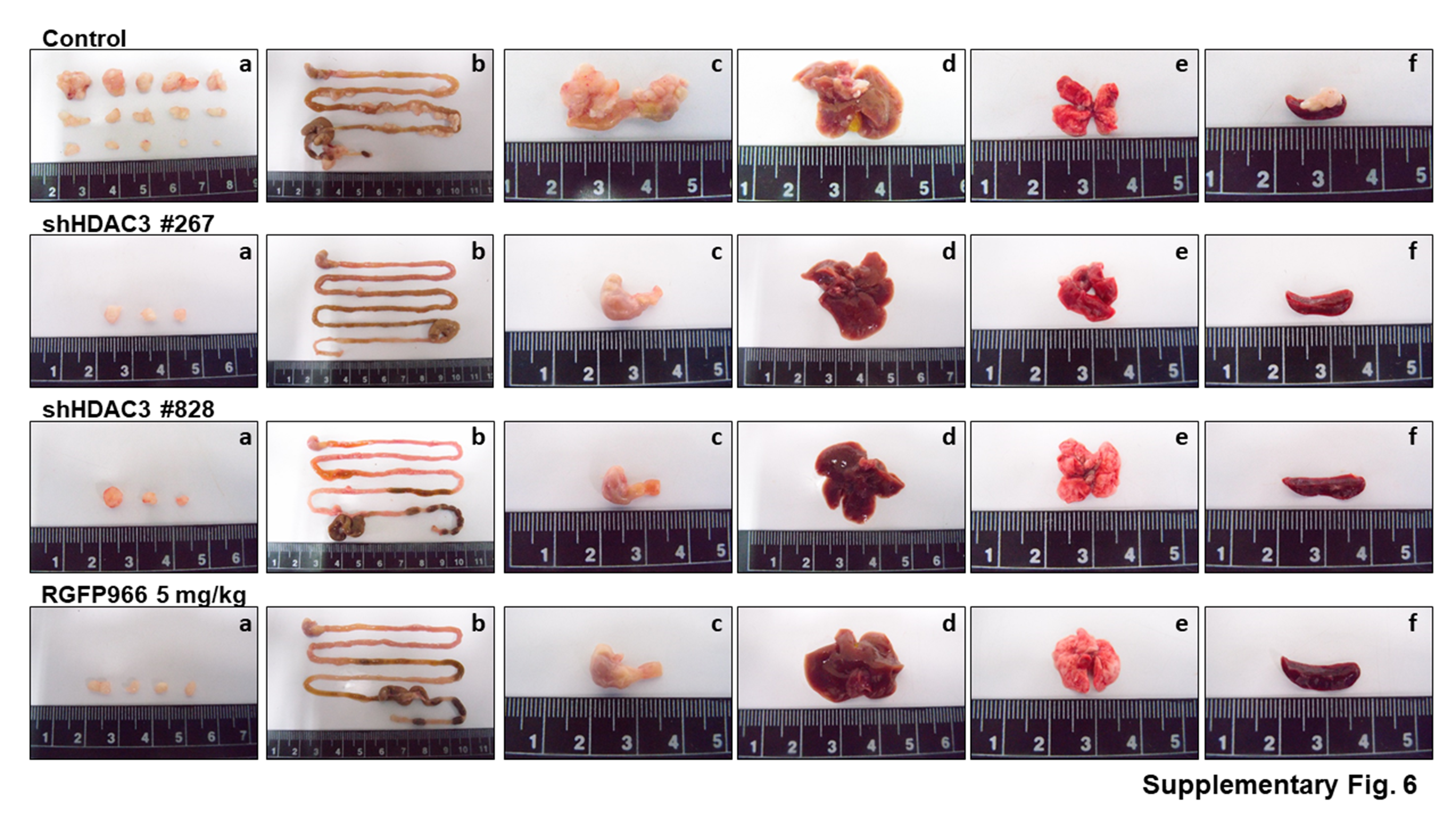

Supplement: Supplementary file 11 — Supplementary Fig. 6 (PNG 25.7 MB) [file 10565_2021_9673_Fig16_ESM.png]

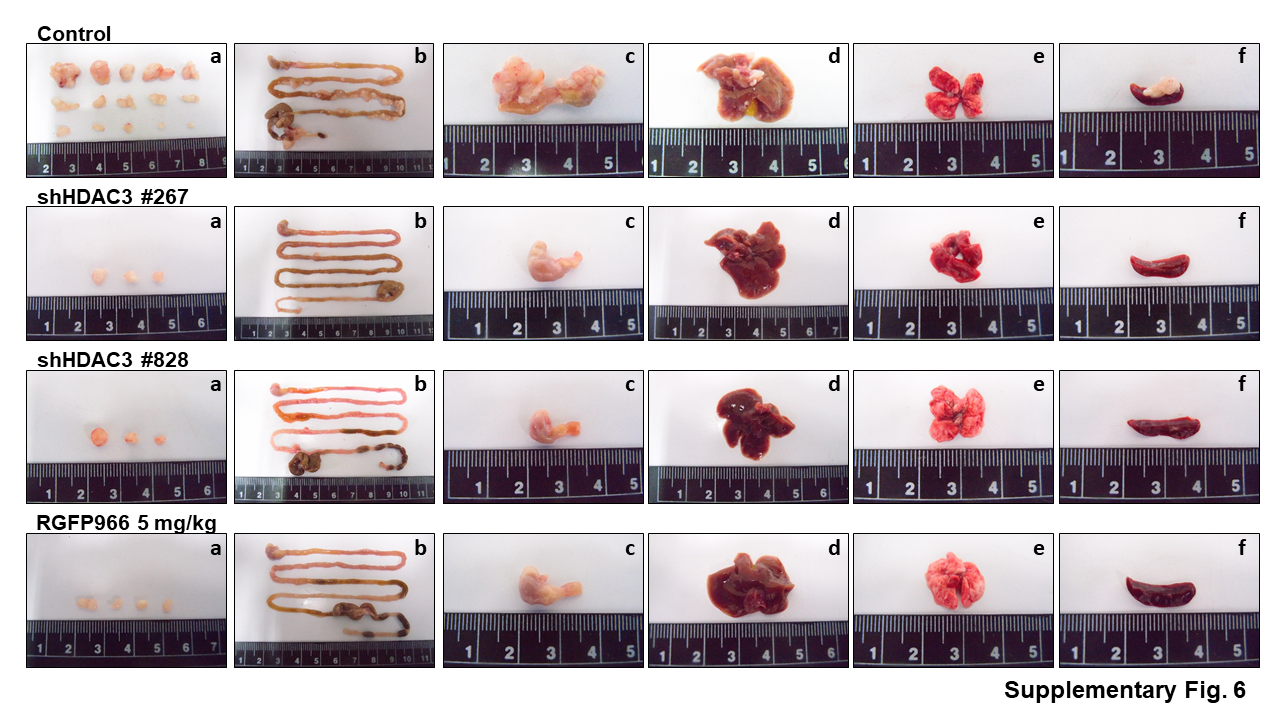

Supplement: Supplementary file 12 — High resolution image (TIF 1200 KB) [file 10565_2021_9673_MOESM6_ESM.tif]

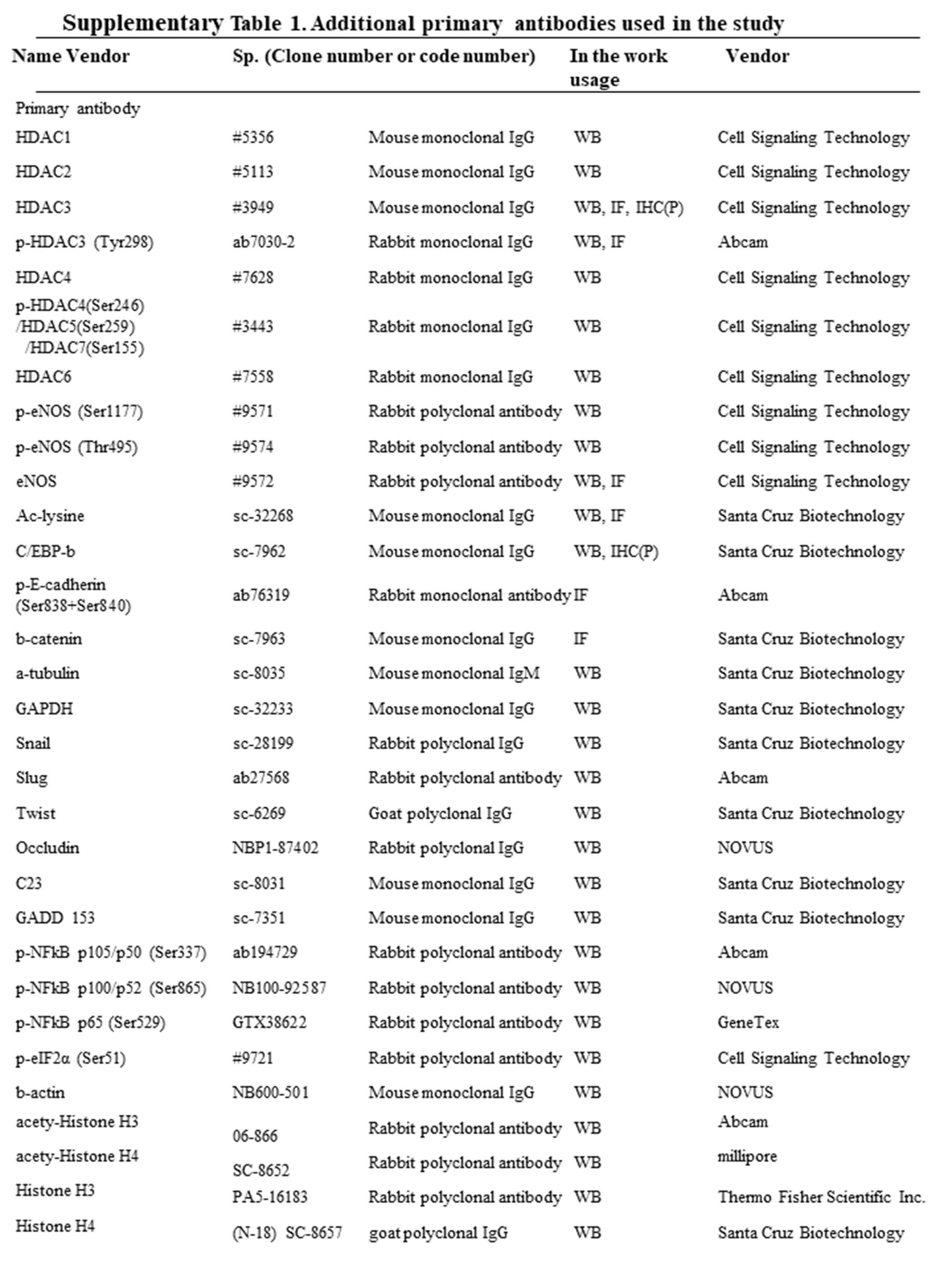

Supplement: Supplementary file 13 — Supplementary Fig. 7 (PNG 19.3 MB) [file 10565_2021_9673_Fig17_ESM.png]

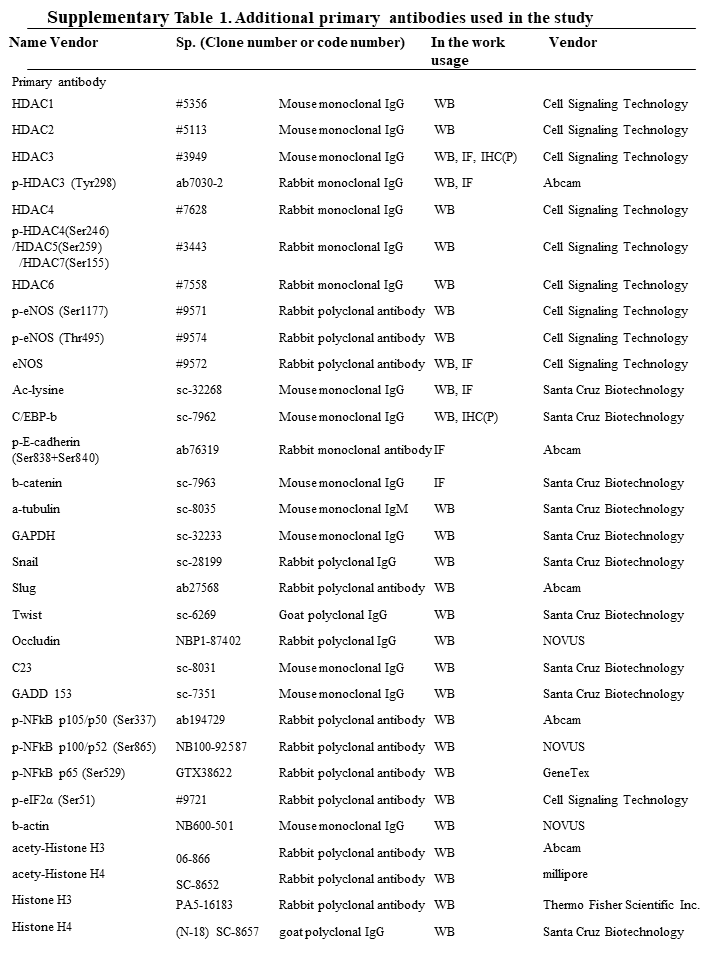

Supplement: Supplementary file 14 — High resolution image (TIF 151 KB) [file 10565_2021_9673_MOESM7_ESM.tif]

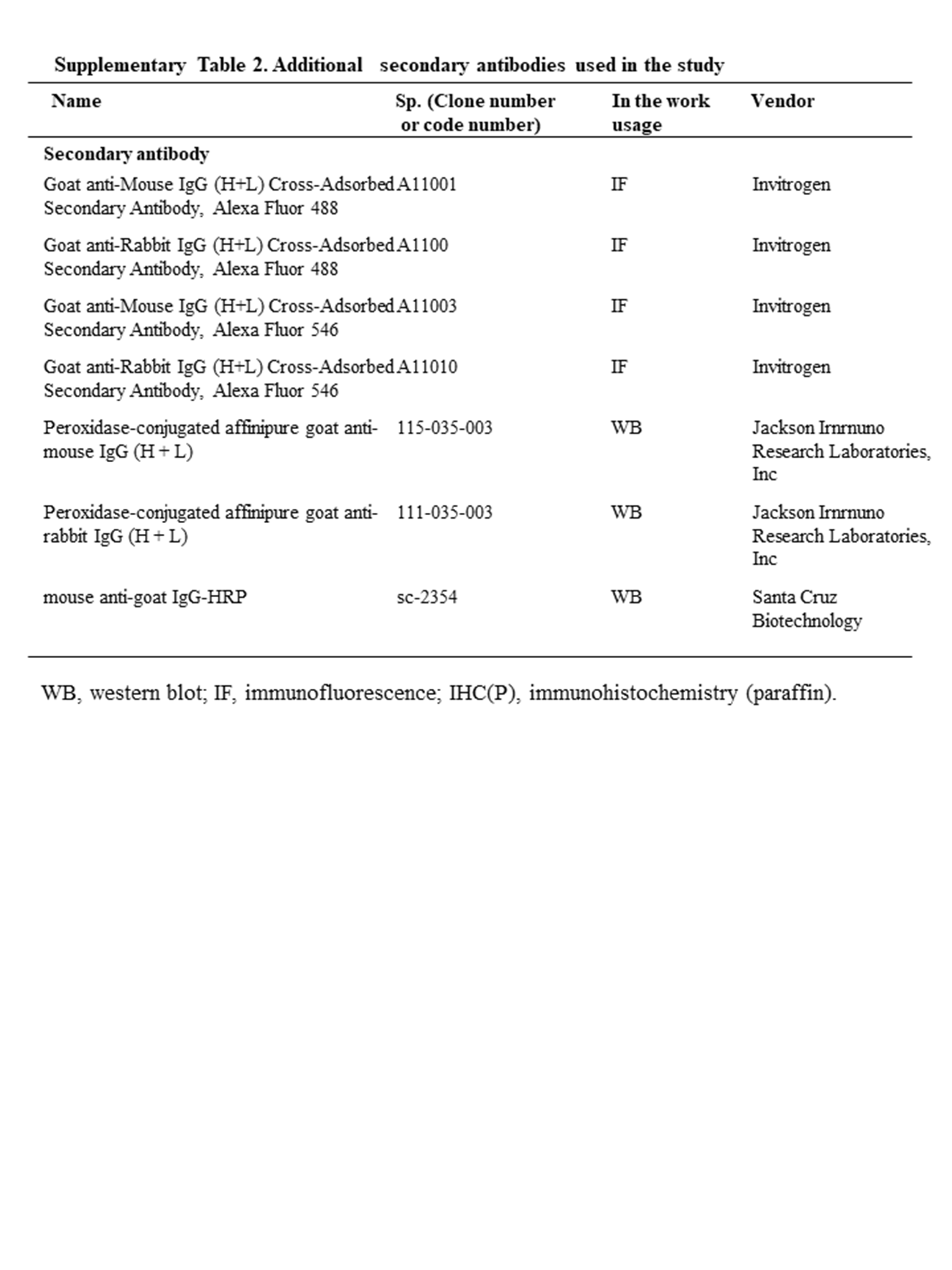

Supplement: Supplementary file 15 — Supplementary Fig. 8 (PNG 19.3 MB) [file 10565_2021_9673_Fig18_ESM.png]

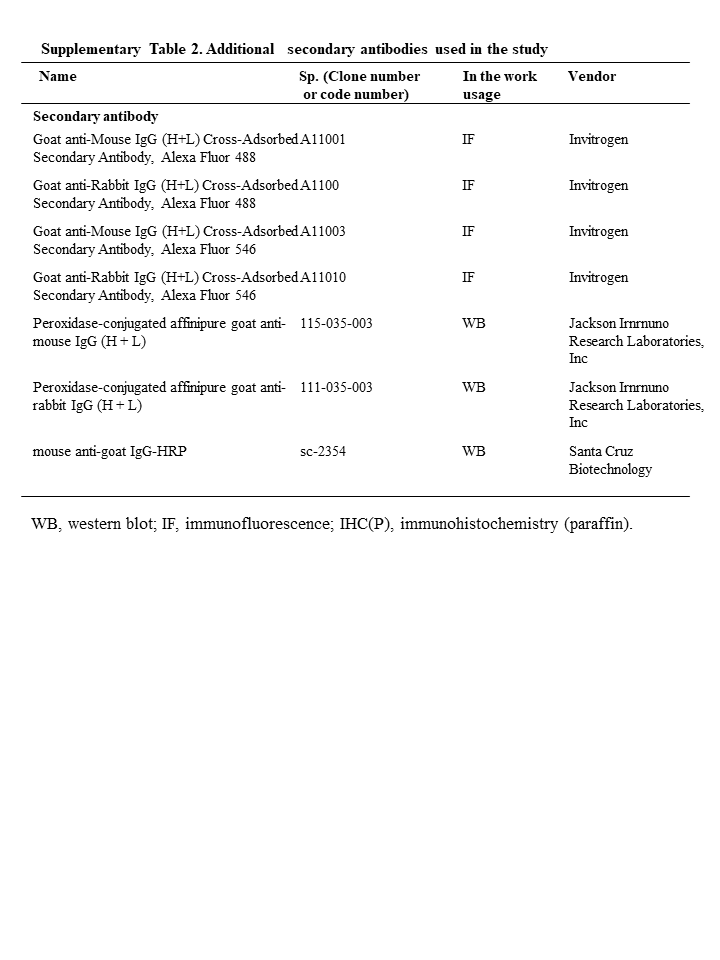

Supplement: Supplementary file 16 — High resolution image (TIF 89 KB) [file 10565_2021_9673_MOESM8_ESM.tif]
